# Lineage-Specific Evolutionary Histories and Regulation of Major Starch Metabolism Genes during Banana Ripening

**DOI:** 10.3389/fpls.2016.01778

**Published:** 2016-12-02

**Authors:** Cyril Jourda, Céline Cardi, Olivier Gibert, Andrès Giraldo Toro, Julien Ricci, Didier Mbéguié-A-Mbéguié, Nabila Yahiaoui

**Affiliations:** ^1^CIRAD, UMR AGAPMontpellier, France; ^2^CIRAD, UMR PVBMTSaint-Pierre, France; ^3^CIRAD, UMR QUALISUDMontpellier, France; ^4^CIRAD, UMR QUALISUDJakarta, Indonesia; ^5^CIRAD, UMR QUALISUDCapesterre-Belle-Eau, France

**Keywords:** starch, evolution, gene duplication, *Musa acuminata*, fruit, amylases

## Abstract

Starch is the most widespread and abundant storage carbohydrate in plants. It is also a major feature of cultivated bananas as it accumulates to large amounts during banana fruit development before almost complete conversion to soluble sugars during ripening. Little is known about the structure of major gene families involved in banana starch metabolism and their evolution compared to other species. To identify genes involved in banana starch metabolism and investigate their evolutionary history, we analyzed six gene families playing a crucial role in plant starch biosynthesis and degradation: the ADP-glucose pyrophosphorylases (AGPases), starch synthases (SS), starch branching enzymes (SBE), debranching enzymes (DBE), α-amylases (AMY) and β-amylases (BAM). Using comparative genomics and phylogenetic approaches, these genes were classified into families and sub-families and orthology relationships with functional genes in Eudicots and in grasses were identified. In addition to known ancestral duplications shaping starch metabolism gene families, independent evolution in banana and grasses also occurred through lineage-specific whole genome duplications for specific sub-families of *AGPase, SS, SBE*, and *BAM* genes; and through gene-scale duplications for *AMY* genes. In particular, banana lineage duplications yielded a set of *AGPase, SBE* and *BAM* genes that were highly or specifically expressed in banana fruits. Gene expression analysis highlighted a complex transcriptional reprogramming of starch metabolism genes during ripening of banana fruits. A differential regulation of expression between banana gene duplicates was identified for *SBE* and *BAM* genes, suggesting that part of starch metabolism regulation in the fruit evolved in the banana lineage.

## Introduction

Starch is the main storage carbohydrate in plants, synthesized in both leaves and non-photosynthetic storage organs of plants. Starch storing organs in economically important crop plants include potato tubers, cereal seeds and also banana fruits which are a major staple food in many subtropical and tropical countries. Bananas are produced by parthenocarpic, mainly triploid hybrids derived from the species *Musa acuminata* of the Zingiberales monocotyledon order, sometimes combined with *M. balbisiana*. An important starch amount of up to 74–88% of dry weight is accumulated during banana green fruit development (Gibert et al., 2009). In dessert bananas, the onset of ripening is characterized by a rapid ethylene production and an increased respiration burst (Burg and Burg, 1965; Liu et al., 1999). During the ripening process, the accumulated starch is almost completely converted into soluble sugars, mostly sucrose, through a fast and efficient enzymatic process (Marriott et al., 1981; Beaudry et al., 1989; Cordenunsi and Lajolo, 1995). Major enzymes responsible for starch biosynthesis and degradation are encoded by multigenic families. They are divided in sub-families that mostly evolved from ancient duplications in the lineages derived from plastid endosymbiosis, with an increasing complexity at the emergence of Chloroplastidae or green lineage. This evolution was accompanied by functional specialization (Ball and Morell, 2003; Deschamps et al., 2008; Cenci et al., 2014; Nougué et al., 2014). In angiosperms, subsequent duplications resulted in increased isoform numbers (Fulton et al., 2008; Georgelis et al., 2008; Nougué et al., 2014). Genes encoding starch metabolism enzymes have been studied mainly in *Arabidopsis*, potato and for monocots mostly in grass species. Little is known about these genes and their evolution in other monocots with starch-storing organs such as bananas.

Starch is composed of two polymer fractions, amylopectin and amylose. They are formed by glucose molecules linked by linear α-1,4 bonds with interspersed α-1,6 bonds that form branching points. Amylopectin, the major component of starch represents typically 75% of starch and it is more branched than amylose. Its molecular structure allows its packing in crystalized arrays, leading to the formation of the starch granule (Zeeman et al., 2010). Amylose is synthesized in the matrix built by amylopectin within the starch granule (Denyer et al., 2001; Zeeman et al., 2010).

Adenosine diphosphoglucose (ADP-glucose), the glucose donor for starch synthesis (Recondo and Leloir, 1961) is synthesized by ADP-glucose pyrophosphorylase (AGPase; EC 2.7.7.27) that uses adenosine triphosphate (ATP) and glucose-1-phosphate as substrates. AGPases are heterotetrameric enzymes composed by two small sub-units (AGPS) and two large subunits (AGPL). In *Arabidopsis thaliana*, they are encoded by a small gene family with tissue-specific genes for AGPL subunits (Lin et al., 1988a,b; Crevillen et al., 2005). The reaction catalyzed by AGPases corresponds to the rate-limiting step in starch biosynthesis (Stark et al., 1992). In addition to AGPase, sucrose synthase has also been proposed to produce ADP-glucose linked to starch biosynthesis (Baroja-Fernandez et al., 2003, 2012; Bahaji et al., 2014).

The starch biosynthesis process *per se* requires activities of three different enzymes (Supplementary Figure S1) namely (i) starch synthases (SS), (ii) starch branching enzymes (SBE) and (iii) starch debranching enzymes (DBE) that can be further divided into two types: isoamylases (ISA) and pullulanases/limit dextrinases (LDA) (Jeon et al., 2010; Zeeman et al., 2010). Starch synthases catalyze the transfer of glucose from ADP-glucose to non-reducing ends of glucose chains via α-1,4-bonds. They are divided based on their amino acid sequences into four sub-families of soluble SSs (SSI, SSII, SSIII, and SSIV) and one sub-family of granule-bound starch synthases (GBSS) (Patron and Keeling, 2005; Leterrier et al., 2008). GBSS is bound to starch granules and synthesizes very long glucan chains that are found mainly in amylose (Tsai, 1974; Denyer et al., 2001; Szydlowski et al., 2011). Synthesis of amylopectin requires the elongation of glucan chains by SSI-IV soluble starch synthases (Delvallé et al., 2005; Szydlowski et al., 2009; Pfister et al., 2014) and the introduction of branch points by SBE enzymes. SBE are α-1-4-glucan-6-glycosyltransferases that cleave α-1,4-bonds in a glucan chain and reattach cut segments to another glucan chain via α-1,6-bonds (Streb and Zeeman, 2012). In addition to SBE, ISA debranching enzymes also have a role in amylopectin synthesis (Hussain et al., 2003). Of the three plant ISA sub-families, ISA1 and ISA2 are proposed to be involved in the removal of misplaced amylopectin branches thus facilitating formation of the semi-crystalline structures observed in starch granules (Wattebled et al., 2005; Streb et al., 2008; Streb and Zeeman, 2014).

Starch breakdown results in the production of maltose and glucose that are further converted to sucrose and it is dependent on the phosphorylation status of glucans at the surface of starch granules (Streb and Zeeman, 2012). In plant chloroplasts, hydrolysis of α-1,4-linked glucose chains is mainly performed by exo-acting β-amylases (BAM), that release β-maltose from exposed non-reducing ends of chains (Scheidig et al., 2002, reviewed by Zeeman et al., 2010). Endo-acting α-amylases (AMY) hydrolyze α-1,4-linked glucose chains and release a mixture of soluble linear and branched oligosaccharides. In addition to amylases, the ISA3 debranching enzyme and in a more minor way, a LDA were found to be necessary to the complete breakdown of branched amylopectin chains in *A. thaliana* (Wattebled et al., 2005; Delatte et al., 2006; Streb et al., 2012).

In banana, cDNAs corresponding to starch metabolism genes have been cloned from ripening fruits. A *GBSS* gene, highly expressed in banana pulp, was found strongly repressed during ethylene-induced ripening (Medina-Suarez et al., 1997). A *BAM* gene (Medina-Suarez et al., 1997; do Nascimento et al., 2006), an *AMY* gene (Junior et al., 2006) and an *ISA3* gene were also found expressed during banana fruit ripening (Bierhals et al., 2004). The availability of the *M. acuminata* reference genome sequence (D’Hont et al., 2012) offers the opportunity to perform a genome-wide analysis of starch gene families and to study their evolution patterns within Commelinids, the monocotyledon clade that includes Arecales (palms), Zingiberales and Poales. Independent whole genome duplications events (WGD) have shaped the genomes of different Commelinid lineages. Three WGDs (named α, β, γ) were found in the banana lineage, one in the palm lineage and two (named ρ, σ) in Poaceae (Tang et al., 2010; D’Hont et al., 2012; Singh et al., 2013). In Poaceae, the maize genome underwent an additional more recent tetraploidy event (Blanc and Wolfe, 2004). Within monocotyledons, duplications of starch metabolism genes through lineage-specific WGD were described for grass species of the order Poales (Wu et al., 2008; Comparot-Moss and Denyer, 2009). They resulted in functional specialization for several starch gene families, mostly through emergence of endosperm-specific genes (Dian et al., 2005; Comparot-Moss and Denyer, 2009). Little is known about the evolution of these gene families in other Commelinid species such as banana. Here, we identified in 12 plant species, all members of six gene families involved in starch metabolism: AGPases, SS, SBE, DBE, AMY and BAM. We studied their evolution with a focus on the banana lineage compared to grasses and we analyzed their expression profiles in banana fruits.

## Materials and Methods

### Identification and Phylogenetic Analysis of Starch Metabolism Genes

Members of starch metabolism gene families were identified using predicted proteomes of twelve plant species (Supplementary Table S1): *M. acuminata* (Musaceae, order Zingiberales); rice, *Brachypodium*, sorghum and maize (Poaceae, order Poales); date palm (Arecaceae, order Arecales), *Arabidopsis*, grapevine, tomato, potato, peach and woodland strawberry. Protein sequences were identified by combining a BLASTP clustering strategy using a list of reference proteins (Supplementary Table S2) and information from Pathway tools databases (Karp et al., 2002), MusaCyc^1^ (Droc et al., 2013), the Greenphyl database^2^ (Rouard et al., 2011) and InterProScan (Quevillon et al., 2005) to confirm and complement clustering results.

Protein sequences were aligned with MAFFT version 6.717b (Katoh et al., 2009). Maximum-likelihood phylogenetic analysis was performed with PhyML version 3.0 (Guindon et al., 2010) using the LG evolution model and gamma distributed substitution rates. The WAG model (Whelan and Goldman, 2001) was used for the BAM family. Tree topology was built based on best of nearest neighbor interchange and subtree pruning and re-graphing methods. An approximate likelihood-ratio test with a Shimodaira–Hasegawa–like procedure (Guindon et al., 2010) was used to estimate branch supports. Sequences from *Chlamydomonas reinhardtii* (Chlorophytae, green algae), *Physcomitrella patens* (Bryophytae, moss) and *Selaginella mollendorffii* (Plantae, Lycophyta) were retrieved from the Greenphyl database v.4.0^2^ (Rouard et al., 2011). They were used to identify ancestral groups that originated before angiosperm radiation and to root trees. The global phylogenetic tree of AGPases and phylogenetic trees of SBE and ISA gene families were rooted using sequences from the cyanobacteria *Anabaena cylindrica*. Short sequences disrupting phylogenetic analyses were not used. Trees were visualized with FigTree v.1.3.1^3^. AGPase subunit types were identified using a global phylogenetic tree of AGPase proteins and annotation of *A. thaliana*, rice and maize AGPases. For each subunit type, a separate phylogenetic tree was constructed.

### Identification of Gene Duplication Modes

Duplicated gene pairs resulting from banana WGD were identified based on *Musa* ancestral blocks available at http://banana-genome.cirad.fr/dotplot (D’Hont et al., 2012) and in the Plant Genome Duplication Database (PGDD^4^, Lee et al., 2013). WGD gene pairs were identified as deriving from α or β *Musa* WGD using available α and β blocks (Garsmeur et al., 2014). Additional paralogous relationships were detected using SynMap^5^ with default parameters and a 3 to 3 quota-align ratio for banana (Tang et al., 2011). Gene scale duplications corresponding to two consecutive duplicated genes (tandem duplications) and duplicated genes separated by twenty or fewer gene loci (proximal duplications) were detected based on the order of gene identifiers along the chromosomes using an in house-script. Syntenic blocks from the PGDD and published data were used to identify WGD-derived genes for plant species other than banana (Bowers et al., 2003; Tang et al., 2010; Schnable et al., 2011; Consortium, 2012). SynFind^6^ was used for synteny search at specific regions between species.

### Plant Materials and Physicochemical Analyses

Plant materials corresponded to an experimental setting that was previously described in Jourda et al. (2014). Banana fruits of the Cavendish cultivar grown in a banana farm in Guadeloupe, were harvested at 40, 60, and 90 days after flowering (DAF) corresponding to the immature green, early and late mature green stages, respectively (Mbéguié-A-Mbéguié et al., 2007). Five independent banana bunches representing biological replicates were sampled for each developmental stage. Fruits harvested at 90 DAF were treated for 24 h with 10,000 ppm of acetylene, an ethylene analog. They were then stored in ventilated chambers with humidified air at 20°C in a similar way to control untreated fruits. One fruit per bunch was sampled immediately after harvest (T0 condition) and for control and treated samples at 2 days (T3 condition) and 4 days (T5 condition) after treatment.

Carbon dioxide production of the whole fruit, peel color, pulp firmness, peel hardness and fruit weight were measured individually on all selected fruits to evaluate their physiological state (see Supplementary Method S1). For the determination of starch content, the material was grinded with liquid nitrogen after harvest or storage at 20°C with/without acetylene and was stored at -80°C until analysis. Starch determination was performed using 500 mg of banana pulp flour oven-dried at 40°C after thawing and a modified Holm-method (Holm et al., 1986). Starch was hydrolyzed by incubation with Termamyl 120 L heat-stable α-amylase enzyme (1124 U/mg protein, Novo Nordisk, Copenhagen, Denmark) and amyloglucosidase (70 U/mg, Sigma, St. Louis, MO, USA). Then, D-glucose molecules from starch hydrolysis were quantified using an enzymatic colorimetric method with glucose-oxidase (GOD) 252 U/mg and peroxidase (POD) 52 U/mg (Sigma, St. Louis, MO, USA) following the principle:

– GOD reaction: D-glucose + O_2_ + H_2_0 →*G*-Gluconate + H_2_O_2_
– POD reaction: 2 H_2_O_2_ + *ρ*-hydroxybenzoic acid + 4-aminoantipyrine → Quinoneimine dye + 4 H_2_O

A blank was done without sample. Free glucose was estimated separately using 100 mg of banana pulp flour treated with sulfuric acid (5 mM) and amyloglucosidase (70 U/mg, Sigma, St. Louis, MO, USA). D-glucose molecules were quantified by spectrophotometry at 510 nm. Starch content is given on dry weight basis and was computed based on the difference between the two D-glucose estimates (Supplementary Method S1). Statistical analysis was performed with an ANOVA followed by a Tukey’s test.

### Expression Profiling for Banana Starch Metabolism Genes Using Published RNA-Seq Data

Banana RNA-Seq data correspond to fourteen libraries that were produced using the method detailed in the supplementary material of D’Hont et al. (2012). They are available at NCBI SRA under accession number ERP0116302. Illumina reads were mapped onto banana gene models. Expression levels for banana starch metabolism genes were normalized in RPKM (reads per kilobase of exon per million mapped reads on banana gene models) and visualized using the MeV application (version 4.9) from the TM4 software suite (Howe et al., 2011).

### RNA Extraction and Quantitative Reverse Transcription Polymerase Chain Reaction (qRT-PCR) Analysis

Total RNA was extracted from 600 mg of pulp tissue corresponding to the median part of the banana fruit as previously described in Jourda et al. (2014). After treatment of RNA with RQ1 DNAse (Promega, Madison, WI, USA), synthesis of first-strand cDNA was performed using 1 μg RNA and SuperScript^®^ III reverse transcriptase (Invitrogen^TM^, Carlsbad, CA, USA). Specificity of designed primers was checked by amplicon sequencing and melting-curve analysis. All selected primers are listed in Supplementary Table S3. The qRT-PCR experiments were performed in two technical duplicates, for four biological replicates per condition, using a Light Cycler^®^ 480 system (Roche Applied Sciences, Switzerland). The banana *MaActin2* (GSMUA_Achr1G05990_001) was used as a reference gene. Computations of normalized transcript abundance [A = E_target_^(-Cptarget)^/E_reference_^(-Cpreference)^] were performed with LightCycler^®^ 480 SW software version 1.5. After a logarithmic transformation of raw data, statistical analysis was performed with an ANOVA followed by a Tukey’s test.

## Results

### Banana Genes Encoding Large and Small AGPase Subunits Have Different Evolutionary Histories

**FIGURE 1:**
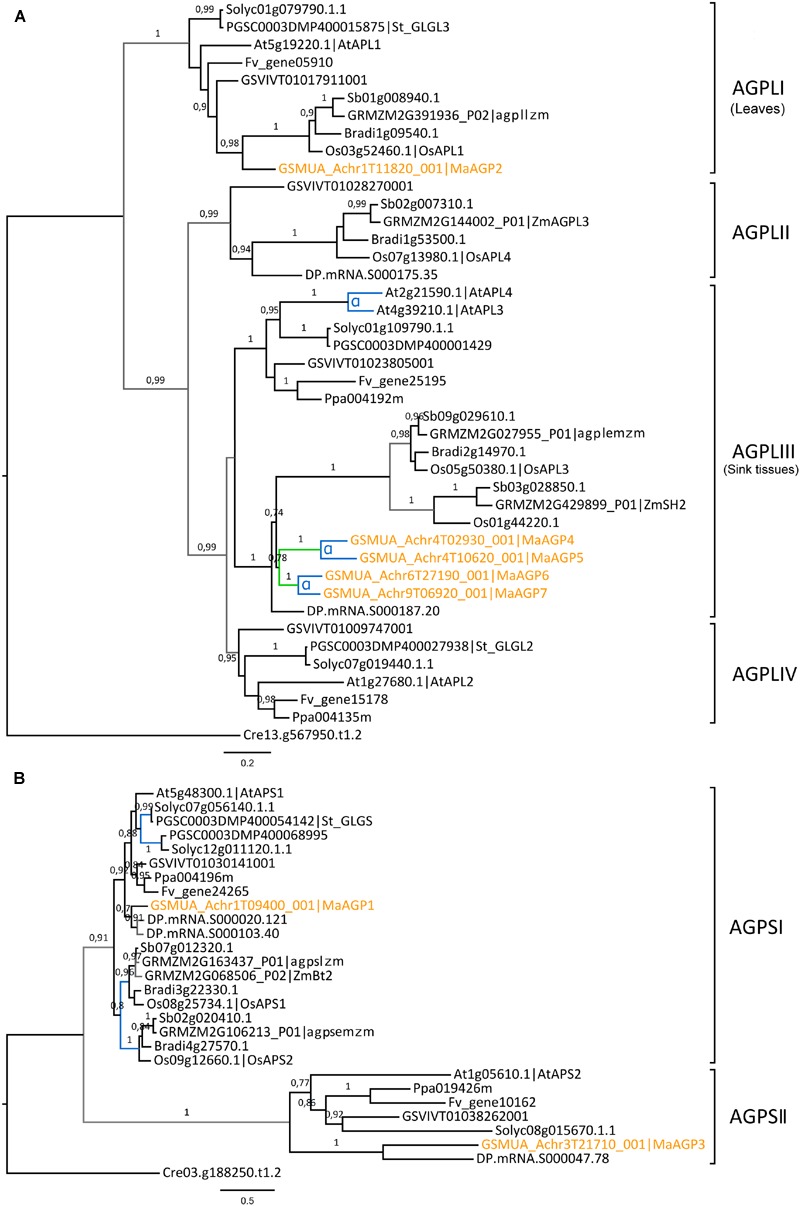
**Phylogenetic trees of the AGPase family. (A)** Maximum likelihood tree of AGPase large subunit predicted proteins (AGPL, 42 sequences). **(B)** Maximum likelihood tree of AGPase small subunit predicted proteins (AGPS, 28 sequences). Trees were rooted using a *Chlamydomonas reinhardtii* (Cre) AGPase sequence. The different subclades are indicated on the right. Branch supports >0.70 (aLRT statistics with SH–like procedure) are indicated. *Chlamydomonas reinhardtii* (Cre), Grapevine (GSVIV), tomato (Solyc), potato (PGSC), woodland strawberry (Fv), *Arabidopsis* (At), peach (Ppa), Brachypodium (Bradi), rice (Os), maize (GRMZM), Sorghum (Sb), date palm (DP) and banana (Ma) identifiers are indicated (in brown for banana). Maize genes nomenclature is based on Huang et al. (2014). Branches are colored according to gene duplication modes: WGD (blue), segmental (green) and unknown (gray). WGD events identified using reconstructed ancestral blocks are indicated (blue letters). Black branches correspond to speciation events. The scale bar represents the estimated amino acid substitutions per site. AGPLI genes are mainly expressed in leaves whereas AGPL genes from other groups are mainly expressed in sink tissues (Corbi et al., 2012).

**FIGURE 2:**
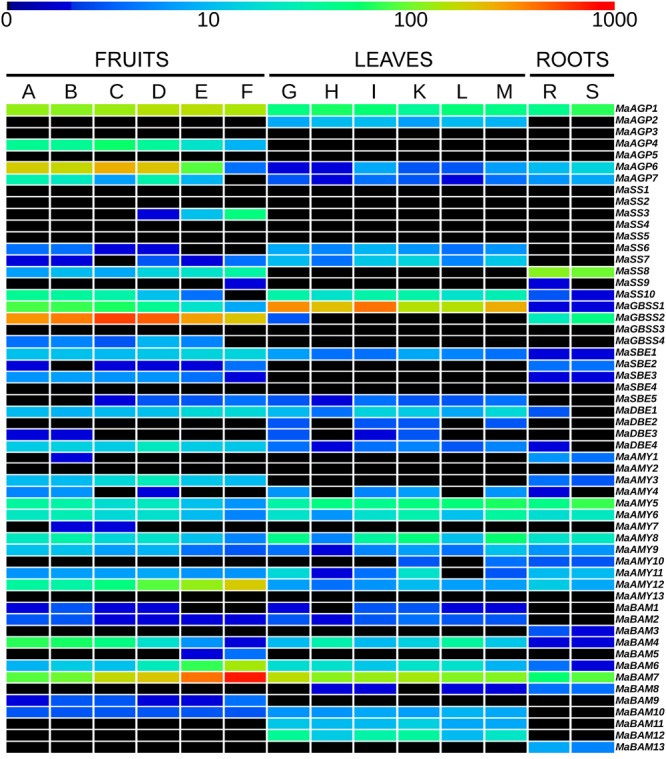
**Expression profiles of starch metabolism genes in banana tissues.** Heatmap visualization of expression levels from published RNA-Seq data (D’Hont et al., 2012). The first six columns correspond to six libraries from Cavendish banana fruits harvested at 40, 60, and 90 days after flowering and stored for 5 days at 20°C without treatment (A, B, C, respectively) or harvested at 40, 60, and 90 days after flowering, treated for 24 h with acetylene and stored for 4 days at 20°C (D, E, F, respectively). The following six columns correspond to six libraries from leaf pieces of banana accessions Pisang Pipit, DH-Pahang and Pisang madu with mock inoculation (G, H, I, respectively) or inoculated with *Mycosphaerella fijiensis* (K, L, M respectively). The last two columns correspond to two libraries from accession Pahang banana roots inoculated with *Fusarium oxysporum* fsp *cubense* (R) or with mock inoculation (S). Transcript abundance was normalized in RPKM (reads per kilobase of exon per million mapped reads on banana gene models) and is indicated with a rainbow color logarithmic scale from dark blue (very weakly expressed) to red (1000 RPKM). Black boxes correspond to genes without detected expression (0 RPKM).

Seven genes encoding AGPases were identified in the banana genome, a number similar to that in other analyzed species (Supplementary Table S4). Five of them encode AGPL subunits and two encode AGPS subunits. Banana AGPL genes were found only in two of the four Angiosperm AGPL sub-families identified by phylogenetic analysis (AGPLI and AGPLIII, **Figure 1A**). These AGPLI and AGPLIII sub-families include known functional AGPL subunits (e.g., AtAPL1, AtAPL3, AtAPL4, agplIzm, ZmSH2, agplemzm; Crevillen et al., 2003; Huang et al., 2014) and have members in all analyzed species. In contrast, AGPLII and IV show gene loss in several lineages (**Figure 1A**). The four AGPL sub-families resulted from angiosperm ancestral duplications (Georgelis et al., 2008; Corbi et al., 2012). Following duplication, one of the gene copies might accumulate mutations leading either to the acquisition of a new function (neo-functionalization) or to the partitioning of the ancestral gene function or gene expression pattern (sub-functionalization) (Force et al., 1999; Lynch and Force, 2000). The most ancestral AGPL duplication, leading to the AGPLI and the AGPLIII groups (**Figure 1A**), was followed by sub-functionalization of paralogs (Corbi et al., 2012). Thus, AGPLI genes are mainly expressed in leaves whereas AGPL genes from remaining sub-families are mainly expressed in sink tissues. Accordingly, expression data indicated that the banana AGPLI gene, *MaAGP2*, was expressed in detached leaf pieces but not in banana fruit and roots (**Figure 2**). In contrast to single copy AGPLI genes, AGPLIII genes were duplicated in several plant lineages including *Arabidopsis*, banana and grasses. In *A. thaliana*, two AGPLIII genes, *AtAPL3* and *AtAPL4* were yielded by the Brassicaceae α WGD (Bowers et al., 2003; Crevillen et al., 2005). The four banana AGPLIII genes (*MaAGP4-7*, **Figure 1A**) are paralogs originating from an ancient segmental duplication followed by the most recent banana WGD (α WGD, **Figure 1A**). The segmental duplication was postulated based on four small syntenic segments including AGPLIII genes and identified with SynMap in the banana genome. These small syntenic segments were not assigned to a *Musa* ancestral block as defined in D’Hont et al. (2012) but it is not excluded that they resulted from WGD. Except for *MaAGP5* which was not expressed in tested conditions, banana AGPLIII genes were mainly expressed in the fruit with different expression levels (**Figure 2**) which is coherent with sub-functionalization of AGPLIII genes in sink tissues. Poaceae AGPLIII genes were also duplicated into two previously described sub-groups (Georgelis et al., 2008; Comparot-Moss and Denyer, 2009; Corbi et al., 2012) corresponding to plastidial AGPases which exist in all plants (e.g., Maïze agplemzm, **Figure 1A**) and to cytosolic AGPases that are found only in cereal endosperm (e.g., maize ZmSH2). The tree topology supports their origin from a grass-lineage duplication as proposed (Comparot-Moss and Denyer, 2009; Corbi et al., 2012).

In contrast to AGPL, AGPS duplicates are few and were mostly described within Monocot or Eudicot lineages (Georgelis et al., 2007, 2008). The AGPS phylogenetic tree revealed two ancestral groups of different size, predating Eudicot/Monocot divergence and each comprising one banana sequence (**Figure 1B**). The largest AGPSI group contains one or two genes per species and corresponds to homologs of the functional *Arabidopsis* AGPS isoform, AtAPS1 (Crevillen et al., 2003). Banana and palm AGPSI sequences were grouped with Eudicot sequences rather than with Poaceae sequences, possibly due to low resolution of AGPSI polymorphisms in analyzed species or higher divergence of Poaceae AGPS genes (Li et al., 2012). Duplicated AGPSI genes were present in Solanaceae, date palm and Poaceae (**Figure 1B**). Functional specialization was described for the two AGPS enzymes resulting from Poaceae WGD (Comparot-Moss and Denyer, 2009): one is plastidial (e.g., agpsemzm, **Figure 1B**), the other cytosolic and endosperm-specific (i.e., ZmBT2 orthologs). In contrast, the absence of duplicated banana genes indicated gene loss after banana WGDs and thus, similar to *Arabidopsis* and several other species (Georgelis et al., 2007), banana has one AGPSI gene that is expressed in different sampled tissues (*MaAGP1*, **Figure 2**).

The AGPSII group (seven sequences, **Figure 1B**) was not clearly defined in published AGPS phylogenies (Deschamps et al., 2008; Georgelis et al., 2008; Corbi et al., 2012) or was predicted to be Eudicot-specific (Li et al., 2012). Here, it contained a sequence from all sampled species except Poaceae (**Figure 1B**). Some elements suggest a minor role of these genes or a possible pseudogenization process: AGPSII sequences are more divergent (average *p*-distance 0.442) compared to highly conserved AGPSI (average *p*-distance 0.154), the *Arabidopsis* AtAPS2 protein is not active (Crevillen et al., 2003) and banana (*MaAGP3*), tomato and *Arabidopsis* AGPSII genes are very lowly or not expressed in different tissues^7^^,^^8^ (Crevillen et al., 2005; **Figure 2**).

Thus, banana AGPL genes evolved differently from those encoding AGPS subunits with an amplification of AGPLIII genes expressed in sink organs through *Musa* large-scale duplications and one main AGPS gene expressed in source and sink organs. This evolutionary pattern is similar to that in *A. thaliana* but differs from AGPase evolution in Poaceae where retention and sub-functionalization occurred for both AGPS and AGPLIII genes, after lineage-specific duplications.

### Duplications and Expression Profile Divergence for Starch Synthesis Genes in Different Commelinid Lineages

The banana reference genome contained 14 predicted genes encoding SSs compared to 9–11 genes per species in grasses and six to eight genes per species in sampled Eudicots (SS and GBSS, Supplementary Table S4). The global SS phylogenetic tree displays the five main sub-families that evolved prior to angiosperm radiation (Patron and Keeling, 2005; Leterrier et al., 2008; Nougué et al., 2014, Supplementary Figure S2). Based on this tree, the banana genome contains four GBSS, one SSI, four SSII, two SSIII and two SSIV encoding genes. The gene GSMUA_Achr1T24640 or *MaSS1* was a fragment of 585 bp nearby *MaSS2*, it was excluded from the analysis and its removal improved branch support values. *MaGBSS3*, one of the four predicted GBSS encoding genes, was fragmented and is likely a pseudogene.

**FIGURE 3:**
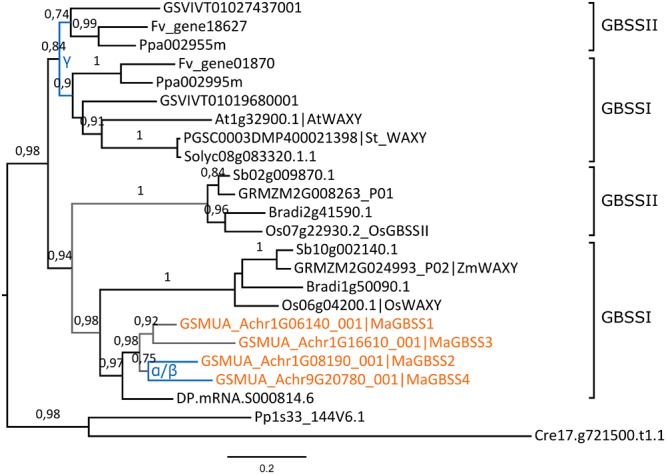
**Expansion of the GBSS family in banana.** The maximum likelihood tree of GBSS predicted proteins was rooted using *Chlamydomonas reinhardtii* (Cre) and *Physcomitrella patens* (Pp) GBSS sequences. Branch supports > 0.70 (aLRT statistics with SH–like procedure) are indicated. Grapevine (GSVIV), tomato (Solyc), potato (PGSC), woodland strawberry (Fv), *Arabidopsis* (At), peach (Ppa), Brachypodium (Bradi), rice (Os), maize (GRMZM), Sorghum (Sb), date palm (DP) and banana (GSMUA) identifiers are indicated (in brown for banana). Branches are colored according to gene duplication modes: WGD (blue) and unknown (gray). Black branches correspond to speciation events. Banana WGD events identified using reconstructed ancestral blocks are indicated. The gamma duplication is inferred based on synteny blocks in PGDD. The scale bar represents the estimated amino acid substitutions per site.

The topology of the GBSS tree suggests independent ancestral duplications yielding GBSSI and GBSSII groups in each of the Eudicot and Monocot lineages (**Figure 3**). Eudicot duplicated *GBSS* genes are present in PGDD on syntenic blocks of grapevine, peach and woodland strawberry and thus could have resulted from the ancestral Eudicot γ hexaploidization (Jaillon et al., 2007). Gene loss likely occurred in some Eudicot lineages since only one *GBSS* gene was found in potato, tomato and *Arabidopsis*. In sampled Commelinids, the phylogenetic analysis grouped together grass *Waxy* genes which determine amylose content in cereal grains (Shure et al., 1983), all four banana *GBSS* genes and one date palm gene. A second GBSS group, GBSSII, comprised here only grass genes (**Figure 3**). The duplication generating these two groups is likely ancestral to Commelinids divergence. The tree topology suggests that all four banana *GBSS* genes have resulted from duplications in the Zingiberales lineage. However, only *MaGBSS2* and *MaGBSS4* were found on banana α/β WGD blocks. Using SynFind, partial synteny relationships were detected only between banana *MaGBSS2*/*MaGBSS4* gene regions and grass GBSSI regions (*ZmWAXY, OsWAXY* and *Sb10g002140*). All together, these results support a common ancestral origin for GBSSI genes but the duplication mode of banana *MaGBSS1* and *MaGBSS3* genes remains unclear.

**FIGURE 4:**
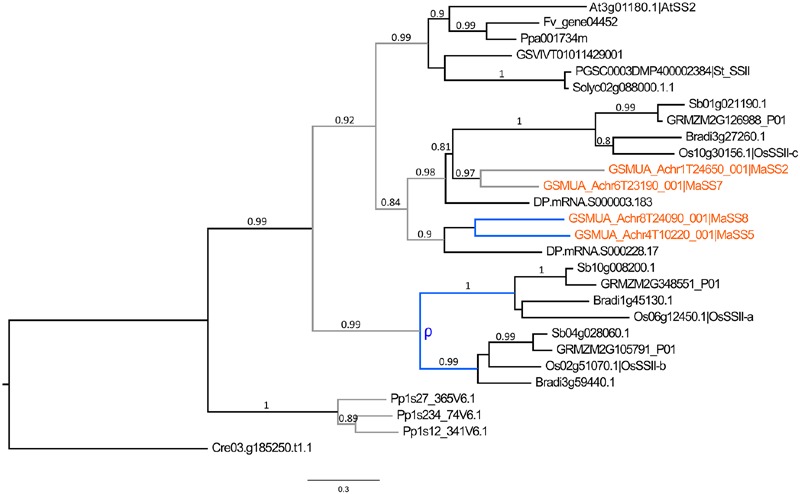
**Phylogenetic tree of SSII predicted proteins.** The maximum likelihood tree of SSII predicted proteins was rooted using a *Chlamydomonas reinhardtii* (Cre) SSII sequence. Branch supports > 0.70 (aLRT statistics with SH–like procedure) are indicated. *Physcomitrella* patens (Pp), Grapevine (GSVIV), tomato (Solyc), potato (PGSC), strawberry (Fv), *Arabidopsis* (At), peach (Ppa), Brachypodium (Bradi), rice (Os), maize (GRMZM), sorghum (Sb), date palm (DP) and banana (GSMUA) identifiers are indicated (in brown for banana). Branches are colored according to gene duplication modes: WGD (blue) and unknown (gray). WGD events identified using reconstructed ancestral blocks are indicated (blue letters). Black branches correspond to speciation events. The scale bar corresponds to estimated amino acid substitutions per site.

The SSI, SSII, and SSIII enzymes are predicted to have distinct roles in the synthesis of short, medium and long chains of amylopectin whereas SSIV is required for initiation of a correct starch granule number (e.g., Commuri and Keeling, 2001; Umemoto et al., 2002; Roldan et al., 2007; Szydlowski et al., 2009). The different SS sub-families showed different patterns of evolution (Supplementary Figure S2). SSI genes and homologs of the functional *AtSS4* gene (SSIV) were mostly single copy in analyzed angiosperm species. The SSII and SSIII genes were found as single copy genes in sampled Eudicots but were duplicated in banana and also, as previously described, in grasses (Wu et al., 2008; Yan et al., 2009; Cheng et al., 2012). In addition, date palm SSII genes were also found duplicated (**Figure 4**). While SSIII duplicates in banana and grasses were derived from large scale duplications specific to each lineage (Supplementary Figure S2), the evolutionary pattern of SSII involved ancestral duplications predating Commelinids divergence in addition to more recent duplications within Commelinid lineages yielding three SII genes in grasses, four in banana and at least two in date palm (**Figure 4**).

**FIGURE 5:**
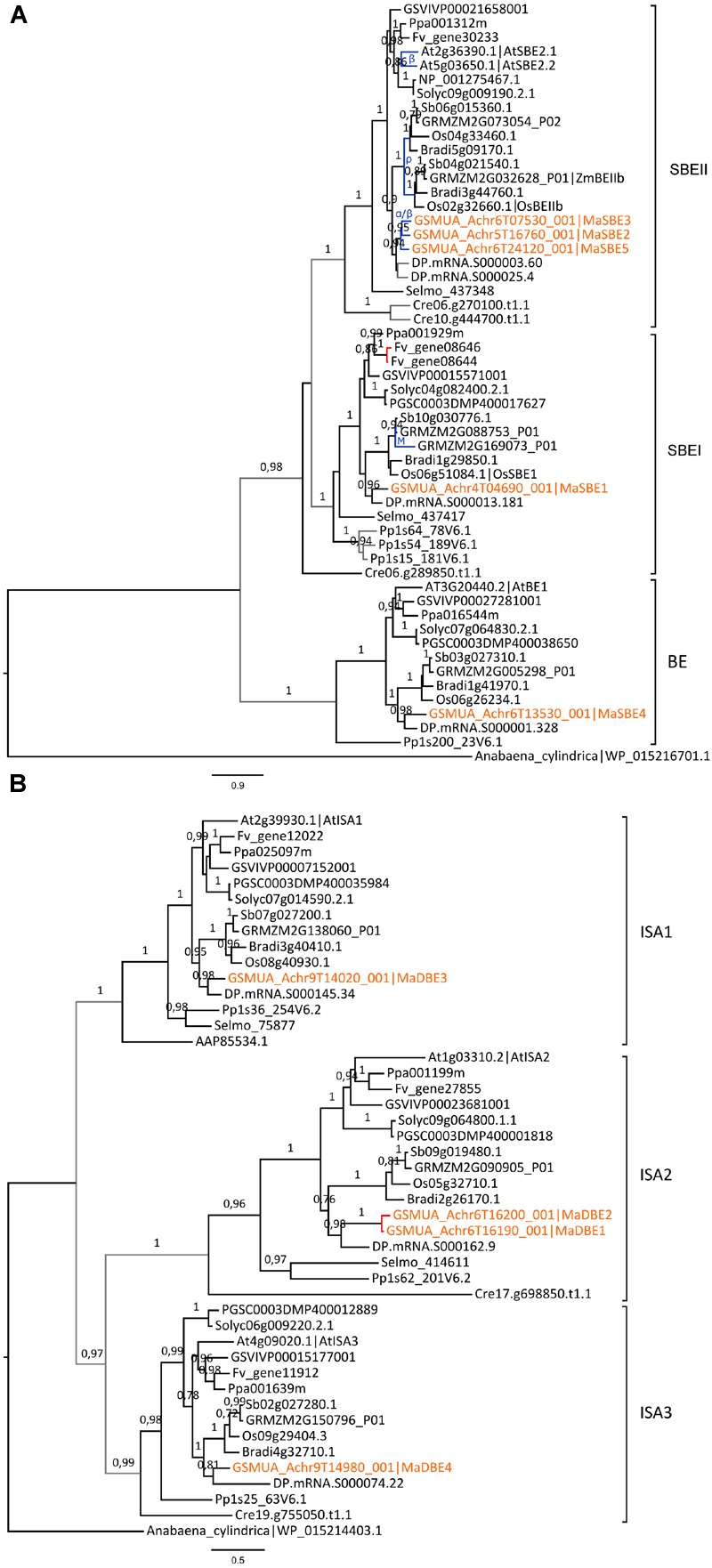
**Phylogenetic trees of branching and debranching enzymes (SBE and DBE) families. (A)** Maximum likelihood tree of SBE predicted proteins. **(B)** Maximum likelihood tree of DBE (ISA) predicted proteins. Trees were rooted using *Anabaena cylindrica* SBE and DBE sequences, respectively. The different subclades are indicated on the right. Branch supports > 0.70 (aLRT statistics with SH–like procedure) are indicated. *Chlamydomonas reinhardtii* (Cre), *Physcomitrella patens* (Pp), *Selaginella moellendorffii* (Selmo), grapevine (GSVIV), tomato (Solyc), potato (PGSC), woodland strawberry (Fv), *Arabidopsis* (At), peach (Ppa), Brachypodium (Bradi), rice (Os), maize (GRMZM), sorghum (Sb), date palm (DP) and banana (GSMUA) identifiers are indicated (in brown for banana). AAP85534.1 and NP_001275467.1 are a *C. reinhardtii* and a potato sequence from Genebank, respectively. Branches are colored according to gene duplication modes: WGD (blue), proximal (red) and unknown (gray). WGD events identified using reconstructed ancestral blocks are indicated (blue letters). Black branches correspond to speciation events. Scale bars represent the estimated amino acid substitutions per site.

Different patterns of evolution were also observed for sub-families of SBEs (**Figure 5A**). Plants possess two types of functional SBE, SBEI, and SBEII, which have distinct roles in the synthesis of long and short chains of amylopectin, respectively (Nakamura, 2002; Satoh et al., 2003). A third group of homologous sequences not known to have a function in starch metabolism (Dumez et al., 2006; Nougué et al., 2014) will not be further considered (BE, **Figure 5A**). The SBEI genes were mostly single copy except for two WGD duplicates in Maize and two tandem duplicates in woodland strawberry (**Figure 5A**). In contrast, two SBEII genes resulting from lineage specific WGDs were present in *Arabidopsis* and grasses as previously shown (Wu et al., 2008; Nougué et al., 2014) and SBEII genes were also duplicated in banana and date palm yielding three and two SBEII genes, respectively (**Figure 5A**).

In the three DBE/Isoamylase DBE sub-families (ISA1 to ISA3), only two ISA2 genes, *MaDBE1* and *MaDBE2*, were predicted as tandem duplicated on banana chromosome 6; the latter being a gene fragment and thus possibly not functional (**Figure 5B**). No gene encoding a pullulanase/limit dextrinase (LDA) was found in the banana reference genome.

In rice, *GBSSI* (*Waxy), SSII-a/SSIII-a* and *SBEIIb* genes were found highly or specifically expressed in reproductive tissues whereas their duplicates were either predominantly expressed in vegetative tissues (*GBSSII, SSII-b*/*SSIII-b*) or steadily expressed in different tissues such as *SSII-c* and *SBEIIa* genes (Hirose and Terao, 2004; Ohdan et al., 2005). The grass *SSII-a/SS-IIb, SSIII* and *SBEII* genes showing expression divergence in the endosperm *vs.* other tissues originated from grass lineage duplications (Wu et al., 2008; Li et al., 2012, **Figures 4** and **5**, Supplementary Figure S2). In analyzed RNA-Seq data for banana, different expression profiles were detected for duplicated genes of the GBSS, SSII and SBEII families (**Figure 2**). *MaGBSS1* (GBSSI), *MaSS7* (SSII), and *MaSBE5* (SBEII) were expressed in both leaves and fruits while *MaGBSS2* (GBSSI), *MaSS8* (SSII), *MaSBE2* and *MaSBE3* (SBEII) were expressed in fruit and roots. In contrast to grasses, these differences of expression profiles in banana were observed here between genes resulting from older duplications of unknown origin while more recent duplicates were either very weakly expressed in available data (*MaGBSS4, MaSS5* and *MaSS2*); or were pseudogenes (*MaGBSS3*). Banana SBEII genes showing different expression profile in leaves and fruits resulted from duplications in the Zingiberales which suggests expression profile divergence in this lineage.

### Expansion of a Specific Sub-family of Amylases through Gene-Scale Duplications in Different Plant Species

**FIGURE 6:**
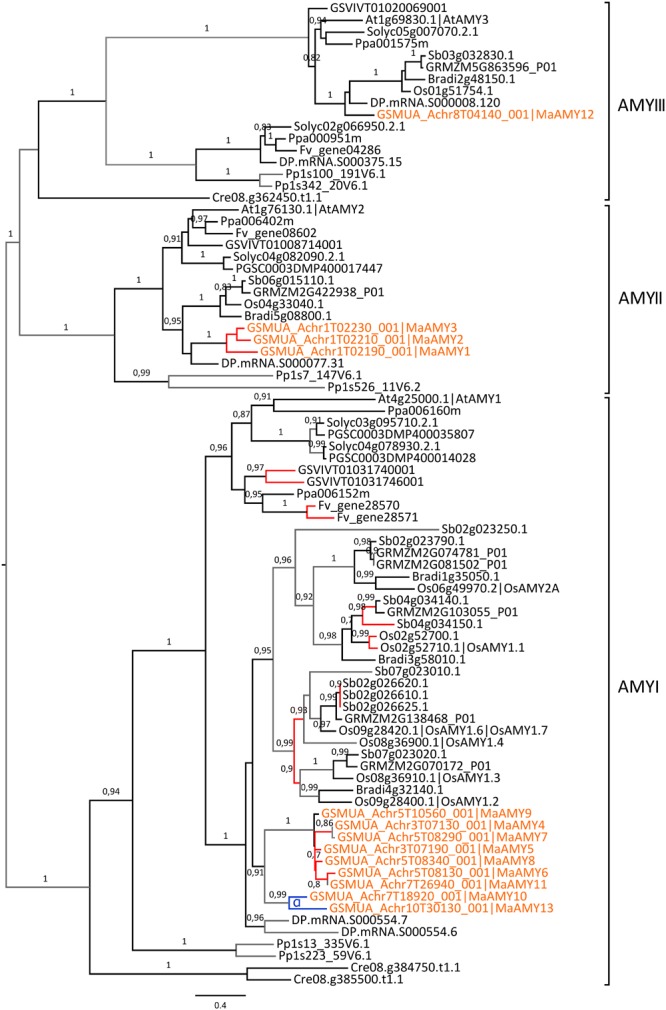
**Acquisition of AMY genes through tandem/proximal duplications.** The maximum likelihood tree of AMY predicted proteins was rooted on midpoint. Branch supports > 0.70 (aLRT statistics with SH–like procedure) are indicated. *Chlamydomonas reinhardtii* (Cre), *Physcomitrella patens* (Pp), grapevine (GSVIV), tomato (Solyc), potato (PGSC), woodland strawberry (Fv), *Arabidopsis* (At), peach (Ppa), Brachypodium (Bradi), rice (Os), maize (GRMZM), sorghum (Sb), date palm (DP) and banana (GSMUA) identifiers are indicated (in brown for banana). Branches are colored according to gene duplication modes: WGD (blue), tandem/proximal (red) and unknown (gray). Black branches correspond to speciation events. WGD events identified using reconstructed ancestral blocks are indicated (blue letters). The scale bar corresponds to estimated amino acid substitutions per site. Sequences were grouped using the tree topology and nomenclature of *Arabidopsis* AMY sequences.

Plant AMY endo-amylases are divided into three phylogenetic sub-families (AMYI-AMYIII, **Figure 6**). One gene of the AMYIII sub-family, *AtAMY3*, was shown to be involved in breakdown of transient starch in *Arabidopsis* leaves (Streb et al., 2012; Seung et al., 2013). The role of AMYII genes is not well known and AMYI genes were mainly characterized in cereals (Stanley et al., 2002, 2005). AMYI genes of cereals encode proteins that are secreted from the scutellar epithelium and aleurone layer into the starchy endosperm where they play a major role in starch breakdown upon seed germination (Asatsuma et al., 2005; Bak-Jensen et al., 2007). The AMYI sub-family shows a striking expansion in grasses particularly in sorghum (9 genes) and rice (7 genes) and also in banana with nine *Musa* AMYI genes (**Figure 6**). In Poaceae, the large expansion of AMYI genes is due to both ancestral and recent tandem duplications and other duplications (**Figure 6**). Among the nine AMYI genes predicted in the banana genome, three corresponded to fragments (*MaAMY4*, 282 bp; *MaAMY7*, 513 bp; *MaAMY11*, 237 bp). The banana AMY1 predicted genes were derived from the *Musa* α WGD (*MaAMY10, MaAMY13*) and five of them resulted from proximal duplications on chromosomes 3 and 5 (*MaAMY4, MaAMY5, MaAMY6, MaAMY7, MaAMY8*, **Figure 6**). These results strongly support that tandem or proximal gene-scale duplications have played an important role in AMYI expansion. In the AMYII sub-family, all species had one gene except banana with a tandem cluster of three genes on chromosome 1 (*MaAMY1, MaAMY2, MaAMY3*). Finally, most analyzed species, including banana (*MaAMY12*), had one AMYIII member orthologous to the *Arabidopsis AtAMY3* gene. The banana AMYIII gene, *MaAMY12* was expressed in all sampled tissues whereas banana AMYII genes (*MaAMY1* and *MaAMY3)* were expressed in fruits and roots. No clear tissue-specific profile was identified here for AMYI genes (**Figure 2**).

The second plant amylase gene family, BAM, has evolved from four ancestral sub-families previously described (BAMI-IV, Fulton et al., 2008, Supplementary Figure S3). The 13 predicted banana BAM encoding genes, including a gene fragment present on non-anchored scaffolds (*MaBAM13*), were distributed in all BAM sub-families showing good orthology relationships with genes from other species except within the BAMIV group. This group was not well resolved, possibly due to differential gene loss between lineages. Up to now, BAM genes playing a major role in starch metabolism are members of the BAMII and BAMIII sub-families which comprise the catalytically active β-amylases AtBAM1 and AtBAM3 and a potential regulator of starch degradation, AtBAM4 (Fulton et al., 2008; Monroe et al., 2014). The function of BAMI genes is not clearly known and the two *Arabidopsis* BAMIV genes, *AtBAM7* and *AtBAM8*, are transcription factors playing a role in plant development suggesting that BAMIV genes have other functions than in starch metabolism (Reinhold et al., 2011; Streb and Zeeman, 2012).

**FIGURE 7:**
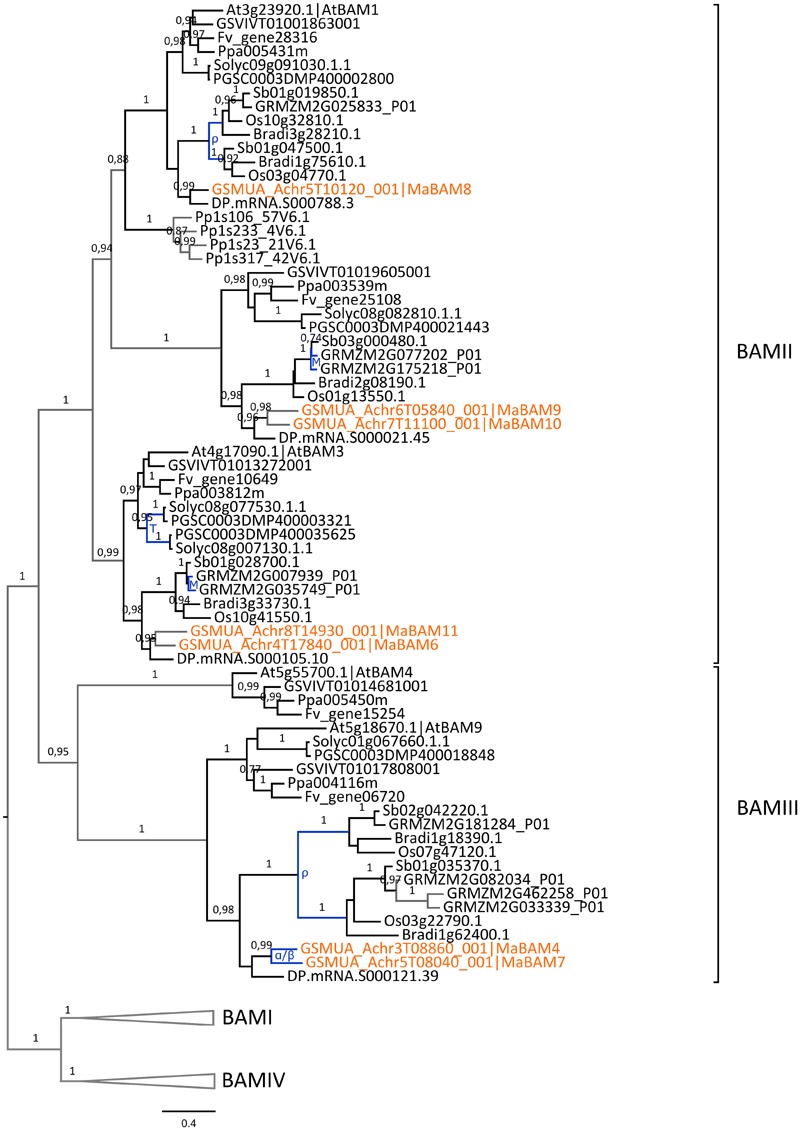
**Phylogenetic analysis of the BAM family.** The maximum likelihood tree of BAM predicted proteins was rooted on midpoint. BAMI and BAMIV sequences are collapsed from the original tree (Supplementary Figure S3). Supports > 0.70 (aLRT statistics with SH–like procedure) are indicated. *Physcomitrella patens* (Pp), grapevine (GSVIV), tomato (Solyc), potato (PGSC), woodland strawberry (Fv), *Arabidopsis* (At), peach (Ppa), Brachypodium (Bradi), rice (Os), maize (GRMZM), sorghum (Sb), date palm (DP) and banana (GSMUA) identifiers are indicated (in brown for banana). Branches are colored according to gene duplication modes: WGD (blue) and unknown (gray). Black branches correspond to speciation events. WGD events identified using reconstructed ancestral blocks are indicated (blue letters). The scale bar corresponds to estimated amino acid substitutions per site. Sequences were grouped using the tree topology and a previous classification (Fulton et al., 2008).

Seven banana BAM genes are members of the BAMII and BAMIII sub-families and could therefore be involved in starch metabolism (*MaBAM4, MaBAM6, MaBAM7, MaBAM8, MaBAM9, MaBAM10, MaBAM11*; Supplementary Figure S3; **Figure 7**).

In contrast to the AMY gene family, the few identified BAM duplications mostly corresponded to WGD and occurred independently in Poaceae, banana and Eudicots (**Figure 7**, Supplementary Figure S3). In the BAMIII sub-family, banana and grass BAMIII gene duplications resulted from banana and grass WGD, respectively. These genes were grouped together with the AtBAM9 Eudicot group rather than with the AtBAM4 group (**Figure 7**). BAMII genes were distributed into three subgroups each comprising Monocot and Eudicot genes with no systematic pattern of duplication. Different expression profiles of *BAM* genes in banana were found for *MaBAM11* (BAMII sub-family) and *MaBAM12* (BAMI sub-family, Supplementary Figure S3) which were expressed in leaves while their respective duplicates were either expressed in all tested tissues (*MaBAM6*) or were weakly expressed in roots (*MaBAM3, MaBAM13*, **Figure 2**).

Thus, gene-scale duplications were a major evolutionary mechanism for *AMY* genes, leading to the expansion of the AMYI sub-family in different species including banana, whereas one or two *BAM* genes per BAM phylogenetic subgroup were observed, often resulting from WGD when duplicated.

### Gene Expression Analysis Identifies Specific Regulations of Starch Metabolism Gene Paralogs Expressed in Banana Pulp

**FIGURE 8:**
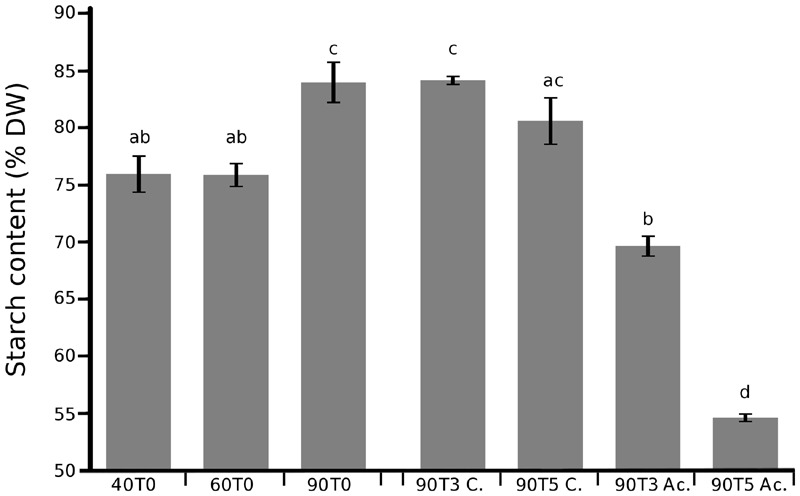
**Accumulation and decrease of starch in banana fruits.** Starch content is expressed in % of dry weight (DW) and was measured individually on fruits harvested at 40, 60, and 90 DAF (40T0, 60T0 and 90T0, respectively) and on fruits harvested at 90 DAF then treated 24 h with acetylene and stored two or four additional days (90T3 Ac. and 90T5 Ac., respectively) and on control fruits not treated and stored three (90T3C.) and 5 days (90T5C.). Three biological replicates were analyzed for each condition. Means, standard errors and different groups from multiple comparisons with confidence interval at 95% (ANOVA followed by Tukey’s test) are indicated.

To better characterize expression profiles of starch metabolism genes in banana fruits, targeted qRT-PCR experiments were carried out on the pulp of dessert banana fruits that were analyzed directly after harvest (T0) at 40, 60, and 90 days after flowering (DAF) and at 2 and 4 days after 24 h of acetylene treatment on fruits harvested at 90 DAF (late mature green ethylene responsive stage, T3 and T5 time points). Different physiological parameters confirmed ripening of 90 DAF fruits treated by acetylene (Supplementary Figure S4). Starch amounts in the pulp showed a relative increase still ongoing in mature green banana fruits between 60 and 90 DAF, and they progressively and significantly decreased after acetylene treatment, with a reduction of around 26% 4 days after treatment (*P* < 0.05, **Figure 8**). This confirmed starch accumulation during banana development and starch breakdown during ripening as previously described for banana (e.g., Bruno Bonnet et al., 2013).

**FIGURE 9:**
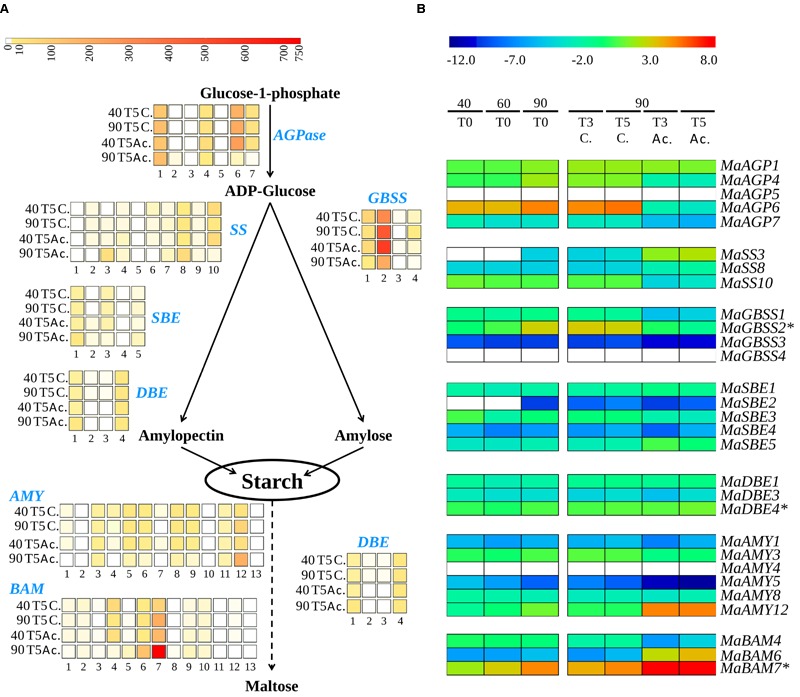
**Expression profiles of starch metabolism genes in banana fruits. (A)** Heatmap visualization of RNA-seq data derived expression levels for the six studied gene families (D’Hont et al., 2012). The four lines correspond to four libraries from banana fruits harvested at 40 and 90 days after flowering and not treated (40T5 C., 90T5 C.) or treated for 24 h with acetylene (40T5 Ac., 90T5 Ac.). RNA-Seq analysis was carried out on RNA extracted 4 days after acetylene treatment (T5) Each column corresponds to a banana gene (see nomenclature in supplementary Table S5). Transcript abundance was normalized in RPKM (reads per kilobase of exon per million mapped reads on banana gene models) and indicated with a color scale from white (not expressed) to red (750 RPKM). The dotted arrow indicates that other enzymes are necessary for the complete conversion. **(B)** Heatmap visualization of relative mRNA abundances from qRT-PCR data. The libraries correspond to cDNA samples from banana pulp tissue. 40T0, 60T0, and 90T0 correspond to fruits harvested at 40, 60, and 90 DAF respectively. 90T3 and 90T5 correspond to fruits harvested at 90 DAF and treated 24h with acetylene (Ac.) or not treated (C.) and stored two or four additional days, respectively. Relative transcript abundance was normalized with the banana Actin2 reference gene and transformed in log2. Gene expression levels are indicated with a rainbow color scale from blue (very weakly expressed) to red (very strongly expressed) and white boxes correspond to genes without detected expression. Stars indicate previously described banana genes.

Genes tested by qRT-PCR were mainly selected based on high expression levels in banana fruits in published RNA-Seq data (>10 RPKM, **Figure 9A**, D’Hont et al., 2012). Their expression in the fruit was confirmed in this independent setting (**Figure 9B**). Absence of expression or low expression levels in the fruit based on RNA-Seq data were also confirmed for seven genes by qRT-PCR (*MaAGP5, MaGBSS3, MaGBSS4, MaSBE2, MaSBE4, MaAMY1, MaAMY4*; **Figure 9**). At least one gene from each of the starch biosynthesis enzyme sub-families was expressed in banana pulp (**Figure 9B**) namely *MaAGP1* (AGPSI), three AGPLIII paralogous genes (*MaAGP4, MaAGP6*, and *MaAGP7*), SS genes *MaSS3* (SSI sub-family), *MaSS8* (SSII) and *MaSS10* (SSIII), *MaGBSS1* and *MaGBSS2*, branching enzyme genes *MaSBE1* (SBEI sub-family), *MaSBE3* and *MaSBE5* (SBEII sub-family), and three DBE/ISA genes. The *MaSS4* and *MaSS9* genes encoding SSIV enzymes were also expressed based on RNA-Seq data (**Figure 9A**) albeit at very low levels. This could be due to the role of SSIV in starch granule initiation that occurs at earlier stages in banana fruit development (Roldan et al., 2007; Crumpton-Taylor et al., 2013; Miao et al., 2014).

Statistical analysis of qRT-PCR data demonstrated differential gene regulation (**Table 1**) leading to different expression profiles across fruit ripening. The AGPase small subunit gene *MaAGP1* and *DBE* genes were stably expressed in all tested conditions. A second set of genes comprised those stably expressed in the green stages or upregulated at the 90DAF stage and then strongly down-regulated after acetylene treatment (**Table 1**, **Figure 9B**). Among them, the genes *MaAGP4, MaAGP6, MaSBE3* and particularly *MaGBSS2* were upregulated between 60 and 90 DAF (**Table 1**), the stage where we clearly observed a high increase of starch content in our setting (**Figure 8**). These genes could be particularly involved in this starch filling stage. The downregulation of expression after acetylene induced ripening occurred for the three functional AGPLIII genes (*MaAGP4, MaAGP6, MaAGP7*), the SSIII gene *MaSS10*, the *MaGBSS2* and *MaGBSS1* genes and *MaSBE3*. Globally, the expression profiles of this second set of genes are consistent with ongoing starch synthesis in the green stages (particularly between 60 and 90 DAF) and a decrease of starch filling during ripening. A third expression profile was unexpected, it corresponded to starch biosynthesis genes that were upregulated after acetylene treatment, namely *MaSS3* (SSI sub-family), *MaSS8* (SSII sub-family) and to a lower extent, *MaSBE1* and *MaSBE5*. *MaSS3* (SSI) is of particular notice as it was induced between 60 and 90 DAF and strongly upregulated during ripening (64 fold, *P* < 0.0001, **Table 1**). If these genes do encode active enzymes at these stages, this suggests the occurrence of some form of starch synthesis after ripening induction, in parallel to the starch degradation process.

**Table 1:** Differential expression of starch metabolism genes in banana pulp.

	Developmental stages			Acetylene treatment		
Gene name	Log2(FC) 60T0	Log2(FC) 90T0	*P*-value	Log2(FC) T3Ac.	Log2(FC) T5Ac.	*P*-value
*MaAGP4*	–	1.86	<0.0001	-3.74	-4.14	<0.0001
*MaAGP6*	–	1.07	0.003	-7.11	-8.44	<0.0001
*MaAGP7*	–	–	n.s	-2.26	-2.82	<0.0001
*MaSS3*	ne	+inf	<0.0001	6.10	6.08	<0.0001
*MaSS8*	–	–	n.s	2.03	2.29	<0.0001
*MaSS10*	–	–	n.s	-4.94	-3.96	<0.0001
*MaGBSS1*	–	–	n.s	-3.90	-2.80	<0.0001
*MaGBSS2*	–	3.53	0.0008	-3.37	-4.44	<0.0001
*MaGBSS3*	–	–	n.s	-4.36	-3.78	<0.0001
*MaSBE1*	–	–	n.s	1.14	–	<0.0001
*MaSBE2*	Ne	+inf	<0.0001	–	–	n.s
*MaSBE3*	–	1.22	<0.0001	-1.51	-2.17	<0.0001
*MaSBE5*	–	–	n.s	–	1.74	<0.0001
*MaBAM4*	–	–	n.s	-4.58	-2.65	<0.0001
*MaBAM6*	–	–	n.s	9.55	9.14	<0.0001
*MaBAM7*	1.14	2.85	<0.0001	3.70	2.70	<0.0001
*MaAMY1*	–	–	n.s	-1.72	–	0.0008
*MaAMY3*	–	–	n.s	-1.83	-1.36	0.0001
*MaAMY5*	-1.36	-3.40	0.0009	-3.35	-2.93	<0.0001
*MaAMY12*	1.08	2.86	<0.0001	4.89	5.08	<0.0001

*log2FC, log2 expression fold change between tested condition and control; 60T0 and 90T0, fruits at 60 and 90 DAF versus fruits at 40DAF; T3Ac. and T5Ac., acetylene-treated 90 DAF fruits at 2 and 4 days after 24 h of acetylene treatment versus untreated controls; n.s., not significant (threshold of two fold and/or *P* < 0.05); ne, not expressed in the two conditions; +inf, gene not expressed in control condition. Only differentially expressed genes are represented.*

As for genes potentially involved in starch degradation, a total of eight *AMY* genes belonging to the three AMY sub-families (*MaAMY1, MaAMY3, MaAMY5, MaAMY6, MaAMY8, MaAM9, MaAMY11*, and *MaAMY12*) and three *BAM* genes (*MaBAM4, MaBAM6*, and *MaBAM7*) of the BAMII and BAMIII sub-families were expressed in banana fruits based on RNA-Seq or qRT-PCR data (**Figure 9**). The amylases-encoding genes tested by qRT-PCR followed five different expression profiles in banana pulp (**Figure 9**, **Table 1**): (i) stably expressed (*MaAMY8*), (ii) stably expressed at the green stages and strongly upregulated during ripening (*MaBAM6*), (iii) progressively upregulated in the 60 or 90 DAF stages and after acetylene treatment (*MaAMY12* and *MaBAM7)*, (iv) stably expressed at the green stages then downregulated during ripening (*MaBAM4, MaAMY1* and *MaAMY3*), (v) downregulated throughout the studied stages (*MaAMY5*). Based on these expression profiles, the three amylases-encoding genes that were strongly upregulated during ripening, *MaAMY12* (AMYIII, 2- ∼30 fold, *P* < 0.0001), *MaBAM6* (500–750 fold, *P* < 0.0001) and *MaBAM7* (6.5–13 fold, *P* < 0.0001) could be particularly involved in starch degradation during banana ripening. *MaAMY12* and *MaBAM6* might encode functional amylases as they are orthologs of *Arabidopsis* genes encoding active enzymes in starch degradation (*AtAMY3* and *AtBAM3*, respectively, Fulton et al., 2008; Seung et al., 2013). The role of *MaBAM7* is more difficult to infer as *MaBAM7* and its banana WGD duplicate *MaBAM4* were assigned phylogenetically to the BAMII sub-family that includes *AtBAM4* a potential regulator of starch degradation and *AtBAM9* of unknown function (Fulton et al., 2008; Monroe et al., 2014). *MaBAM7* and *MaBAM4* have opposite expression profiles after acetylene treatment (**Figure 9**, **Table 1**) and thus represent a case of sub-functionalization by expression profile divergence after banana lineage WGD. Although less striking, different regulation of expression in the fruit for banana WGD duplicated genes was also observed here for *MaSBE3 vs*. *MaSBE5* (**Figure 9**, **Table 1**). This provides an additional element in favor of sub-functionalization of at least a part of starch metabolism genes in the Zingiberales lineage.

## Discussion

Starch metabolism in the fruit is a major agricultural feature of banana as a staple food in tropical and subtropical countries, and a popular fruit worldwide. Based on the banana reference genome sequence (D’Hont et al., 2012), we have identified and classified banana genes from six starch metabolism gene families using comparative genomics and phylogenetic tree reconstructions. Frequent retention of paralogous genes was observed in banana. In several cases, these duplications were associated to the emergence of specific expression patterns, yielding genes highly expressed or differentially regulated in banana fruits. This study also confirms the independent evolution of starch metabolism genes in grasses compared to other Commelinids.

### Independent Duplications of Starch Metabolism Genes in Commelinid Lineages

The banana reference genome and the date palm draft genome sequences offered new perspective on the evolution of gene families within monocotyledons by adding anchor points in addition to grasses. All six gene families analyzed here evolved first from ancestral duplications predating angiosperm divergence which gave rise to their main sub-families (AGPLI-IV, SSI-IV, GBSS, SBEI-III, DBEI-III, BAMI-IV, AMYI-III, Ball and Morell, 2003; Deschamps et al., 2008; Nougué et al., 2014). Gene loss occurred frequently resulting in phylogenetic groups with genes from only few species (e.g., AGPLII, AGPLIV and AGPSII, **Figure 1**). Based on available functional data and expression profiles (this work and e.g., Crevillen et al., 2003; Huang et al., 2014), some of these genes could have highly specialized functions in specific cell types or minor functions, or they might be evolving through a pseudogenization process. In the AGPLI, SSI, SBEI, and all DBE sub-families for which important functional genes for starch biosynthesis were described (Zeeman et al., 2010), no or very few duplicated genes were found despite the occurrence of several WGD events in Angiosperms (**Figures 1A** and **5**, Supplementary Figure S2). This suggests that for these enzymes, selection has favored a single functional gene per species.

In contrast, the AGPLIII, GBSS, SSII, SSIII, SBEII, BAMII, BAMIII and to a lesser extent AGPSI (sub)-families showed further independent duplications in different lineages mainly through large scale duplications whereas AMYI and AMYII were amplified through gene scale duplications in several species. In addition to lineage specific whole genome duplications such as those that occurred in Poales or in Zingiberales, several more ancient WGD events were predicted early in the evolution of land plants. Two of them occurred before monocot-eudicots divergence in the common ancestor of seed plants and in the ancestor of angiosperms (Jiao et al., 2011). In addition, the gamma triplication occurred at, or close to, the origin of eudicots (Jaillon et al., 2007; Jiao et al., 2012) and in monocots, a whole genome duplication named tau and shared by 11 Commelinid genomes has been recently identified (Jiao et al., 2014). Within starch enzyme sub-families, we have found few shared duplications between sampled species from the different Commelinid orders. Palm and banana SSII genes might have been duplicated prior to Commelinid lineage divergence while grass GBSS could have originated from a monocot ancestral duplication (**Figures 3** and **4**). The timing of monocot GBSSI/GBSSII duplication is unclear since our GBSS tree topology differed from previously published ones (Cheng et al., 2012) which suggested a shared ancestral GBSS duplication between eudicots and monocots thus predating eudicot/monocot split. The identification of eudicot GBSSII and GBSSI genes on grapevine syntenic blocks implies that they originated from the gamma hexaploidization (Jaillon et al., 2007) that is at the root of eudicot radiation. In monocots, the tau WGD that is shared by 11 Commelinid genomes (Jiao et al., 2014) could be at the origin of the grass GBSSI/GBSSII duplication or of a banana and palm SSII shared duplication.

Several of the remaining starch gene duplications in Commelinids were attributed to WGD events that occurred within these lineages. Previous studies have described the impact of the grass lineage WGDs on the evolution of starch biosynthesis genes (Wu et al., 2008; Comparot-Moss and Denyer, 2009; Li et al., 2012). The addition of banana and palm gene sequences in our analyses confirmed the emergence of grass-specific starch pathway genes (AGPases, SSII-2 and SSII-3, SSIII and SBEII, Georgelis et al., 2008; Comparot-Moss and Denyer, 2009; Corbi et al., 2012). They originated from grass lineage duplications including the rho WGD that occurred around 70 million years ago before the radiation of major cereal clades (Paterson et al., 2004; Salse et al., 2008; Tang et al., 2010). For banana, we could clearly assign some but not all of the banana lineage duplicated starch genes to ancestral blocks of the alpha and/or beta duplications that were estimated to have occurred in a short time frame around 65 million years ago (D’Hont et al., 2012). In some cases (AGPLIII, SSIII, **Figure 1A**, Supplementary Figure S2), duplicated copies of banana genes were assumed to be derived from a segmental duplication based on small syntenic genome segments that were not attributed to ancestral blocks. These genome segments could also correspond to highly rearranged segments resulting from the three banana lineage WGDs including the gamma WGD that occurred around 100 million years ago (D’Hont et al., 2012). We also identified starch pathway gene duplications in the draft sequence of the date palm genome (Al-Mssallem et al., 2013) but their number might be underestimated and it was not possible to identify their duplication mode. The palm lineage WGD estimated to be 75 million years old (D’Hont et al., 2012; Singh et al., 2013) might be at the origin of some of these genes.

Among the studied gene families, AMYI showed a particular profile of expansion through gene-scale duplications in addition to WGD (**Figure 6**). Increased α-amylases (AMY) copy numbers could result in increased amounts of enzyme necessary for rapid starch breakdown in cereal grains. However, it is difficult to infer a similar hypothesis for banana given that the function of banana AMYI is still unclear. AMYI genes encode secreted enzymes (Asatsuma et al., 2005) and can be induced by biotic and abiotic stress (Doyle et al., 2007). Their amplification by lineage or even species specific tandem duplications could provide with species-specific adaptive templates. On the other hand, their tendency to be duplicated in tandem might also be due to particular features of AMYI sequences or AMYI loci.

### Sub-functionalization of Starch Metabolism Genes in Different Commelinid Lineages

After WGD, most duplicated genes are lost but some are preferentially retained and several models have been proposed to explain their retention (Freeling, 2009). Among them, the neo-functionalization and sub-functionalization models propose that the acquisition of a new function or partitioning of ancestral functions either at the biochemical level or in terms of expression pattern might explain retention of genes after duplication (e.g., Force et al., 1999). An example illustrating these models is the emergence from grass WGDs of a dominant starch synthesis pathway with sub-functionalization of AGPS and AGPLIII duplicates into the cytosol of endosperm cells and the evolution of an ADP-glucose transporter from a duplicate of a plastidial adenine nucleotide transporter named PANT2 (Wu et al., 2008; Comparot-Moss and Denyer, 2009). This allowed the transport of cytosol-synthesized ADP-glucose into plastids, for starch synthesis. It was proposed that a possible selective advantage of this alternate pathway was a more energy efficient synthesis of ADP-glucose in the cytosol compared to plastids (Comparot-Moss and Denyer, 2009). No indications of the existence of a similar cytosolic AGPase-based pathway have been found outside grasses (Beckles et al., 2001). Although banana AGPLIII genes were duplicated, we found that banana has one main AGPS gene expressed in source and sink organs. Based on the evolutionary pattern of banana AGPases, we could not find evidence that an AGPase-based alternate pathway, similar to that in cereals, exists in banana. Three out of four AGPLIII banana genes were expressed in banana pulp and two of them were significantly upregulated between 60 and 90 DAF (**Figure 9**, **Table 1**). There is evidence supporting that synthesis of ADP-glucose by AGPases is a rate-limiting step in starch biosynthesis (Stark et al., 1992). One possible hypothesis for retention of AGPLIII banana genes could be an increased production of ADP-glucose in banana fruit tissues thus favoring high flux starch synthesis. The preservation of duplicates resulting from selection on the absolute abundance of certain gene products was proposed to explain the survival of duplicates from the *Arabidopsis* more ancient WGD (WGD-β, Bekaert et al., 2011). Alternatively, different regulatory properties of AGPL subunits or as is the case for *Arabidopsis* AGPLIII genes, a differential expression profile in specific cell types might also explain the retention of duplicated banana AGPLIII genes (Crevillen et al., 2003, 2005; Georgelis et al., 2009).

Further downstream the starch pathway, the expression patterns of banana duplicates *MaGBSS2, MaSS8* (SSII), *MaSBE2, MaSBE3* suggest sub-functionalization of starch biosynthesis genes in banana sink tissues. For some of these genes (*MaGBSS2 vs. MaGBSS1* and *MaSBE3 vs*. *MaSBE5)*, we could show a specific regulation of expression in the fruit supporting sub-functionalization (**Figure 9**, **Table 1**). Tissue specific expression profiles in banana were observed between the oldest GBSS and SSII duplicates suggesting that sub-functionalization of these genes in fruit/sink tissues could be ancient, possibly before Commelinids divergence (SSII) or early in the evolution of the banana lineage. The opposite regulation of expression during fruit ripening of two *BAM* duplicates (*MaBAM4* and *MaBAM7*, **Figures 7** and **9**, **Table 1**) and also of *SBE* genes resulting from banana WGD (**Figures 5** and **9**, **Table 1**) suggests that part of the regulation of starch metabolism in banana fruits did evolve in the Zingiberales lineage. As for *AMY* duplicated genes, we could not find here indications of sub-functionalization but it is not excluded that it occurs in untested physiological conditions.

### Complex Transcriptional Regulation of Starch Metabolism Genes in Banana Fruits

Up to now, few starch metabolism genes have been identified and analyzed during banana fruit development and ripening (Clendennen and May, 1997; Purgatto et al., 2001; Bierhals et al., 2004; do Nascimento et al., 2006; Junior et al., 2006). Here, we have found that one gene encoding an AGPSI sub-unit (*MaAGP1*) was expressed in banana pulp, together with three AGPLIII genes (**Figure 9**). Members of the AGPSI group are highly constrained (Georgelis et al., 2007; Corbi et al., 2012) possibly because they are less tissue specific and thus must form functional heteromeric enzymes with multiple AGPL (Georgelis et al., 2007). This suggests that in banana fruits, functional heteromeric AGPases could be formed with MaAGP1 and either of the *MaAGP4, MaAGP6* and *MaAGP7* gene products. If protein levels are well correlated to gene expression levels, then the main AGPase heteromeric enzymes in banana pulp might be formed by products of *MaAGP1* and the highly expressed *MaAGP6* and *MaAGP4* genes. At analyzed stages during fruit green mature stages and ripening, differential regulation of expression was observed for genes of the AGPL sub-family but not for the AGPS gene. The constant expression of *MaAGP1* might explain the accumulation of an AGPase small subunit identified by 2D electrophoresis during banana ripening (Toledo et al., 2012). How these profiles do translate in terms of heterotetrameric enzyme amounts and enzymatic activity is not well known in banana and requires additional experiments.

Amylose represents approximately 20% of starch content in banana fruits (Zhang et al., 2005; Dufour et al., 2009) and is synthesized by GBSS (Denyer et al., 2001). In addition, GBSS might also be involved in the synthesis of extra-long unit chains of amylopectin (Hanashiro et al., 2008). A recent study has proposed six banana *GBSS* genes classified in two sub-groups (Miao et al., 2014). Our phylogenetic analysis confirmed the four members of the first sub-group as *GBSS* genes whereas the two remaining genes corresponded to the soluble SSII genes *MaSS5* and *MaSS8*. Among the two *GBSS* genes that we found expressed in banana pulp, the highly expressed *MaGBSS2* corresponds to the *GBSS* gene previously described as induced at the early stage of banana ripening (Xu et al., 2007), and strongly repressed after ethylene treatment (Medina-Suarez et al., 1997). This *GBSS* gene was also found expressed in banana corm but not in the peel and leaves (Clendennen and May, 1997) which supports its major implication in starch synthesis in banana sink tissues. Compared to *MaGBSS1* which was also expressed between 40 and 90 DAF (**Figure 9**), *MaGBSS2* might encode the main GBSS enzyme involved in starch synthesis at the mature green stages.

We have also identified a set of *SS, SBE* and *DBE* genes expressed in banana pulp among which the gene *MaDBE4* corresponds to the previously identified banana ISA3 gene (Bierhals et al., 2004). Overall, starch genes expression profiles were consistent with starch synthesis and filling during the green stages and a decline of starch synthesis throughout acetylene-induced ripening. Starch synthesis during the green stages seems to rely mainly on GBSS, SSII, SSIII and SBE genes as banana SSI gene expression was undetectable particularly between the early and immature green stages (**Figure 9**). The SSI enzyme plays an important role for transient starch synthesis in *Arabidopsis* leaves (Delvallé et al., 2005) but the SSI gene is lowly expressed and its downregulation had no apparent impact on amylopectin synthesis in potato tubers (Kossmann et al., 1999). It is therefore possible that this enzyme is not crucial for banana starch synthesis at this stage or that its function is compensated by other enzymes. The up-regulation of two *SS* genes and two *SBE* genes after acetylene treatment suggests that a form of starch synthesis is ongoing after ripening induction, if active enzymes are indeed encoded by these genes at these stages. Similar expression profiles were observed in ripening grapevine for three transcripts encoding putative SS enzymes (Lijavetzky et al., 2012) although starch content also decreases during grape ripening. One possible explanation for the induction of *SS* and *SBE* genes could be related to high soluble sugar levels produced by starch degradation. Experiments based on external supply of glucose to banana undergoing starch breakdown detected concomitant starch synthesis and led to the hypothesis that if the hexose monophosphate pool resulting from starch degradation becomes too large, part of it could be diverted back into starch synthesis (Hill and Rees, 1993; Hill and Rees, 1995).

In ripening banana fruits, an increase in BAM activity was shown to be dependent on exposure to ethylene and correlated to a high rate of starch degradation (do Nascimento et al., 2006). When an inhibitor of ethylene signaling was used, some starch degradation did occur but at a slower rate (do Nascimento et al., 2006). This indicated a high dependence on the de-polymerization activity provided by BAMs but also that other enzymes were likely to be involved in the degradation process. Our gene expression analysis was extended to members of three major gene families involved in starch degradation (AMY, BAM and DBE). Among the seven *AMY* genes found here expressed in banana fruits, the gene *MaAMY8* was previously published as being expressed during banana ripening (Junior et al., 2006). *MaAMY8* is a member of the AMYI sub-family to which belong several cereal grain AMYs (Stanley et al., 2002, 2005). As AMYI enzymes are generally predicted to be secreted (Stanley et al., 2002, 2005), a role of these enzymes in banana starch degradation remains to be determined. In *A. thaliana*, the AMYIII gene, *AtAMY3*, has been shown to perform efficient starch degradation in *Arabidopsis* chloroplasts in synergy with AtBAM1 (Seung et al., 2013). In banana fruits, it is therefore possible that *MaAMY12*, the *AtAMY3* orthologous gene is involved in starch degradation together with BAM and ISA3 genes. Upregulated only after acetylene treatment, *MaBAM6* is the ortholog of *AtBAM3* and of potato *CT-BMY* which encode dominant catalytically active isoforms in starch degradation (Scheidig et al., 2002; Kaplan and Guy, 2005; Fulton et al., 2008). *MaBAM6* might encode a catalytically functional enzyme that contributes to an increased BAM activity which is dependent of ethylene signaling (do Nascimento et al., 2006). In addition to *MaBAM6*, we have found that two additional BAM genes, *MaBAM4* and *MaBAM7*, were expressed in banana pulp with different expression regulation patterns before and after acetylene-induced ripening (**Figure 9**, **Table 1**). One of them, *MaBAM7*, corresponds to the BAM-encoding gene (Z93112) identified as being highly expressed in ripening banana fruits (Medina-Suarez et al., 1997; do Nascimento et al., 2006). The close phylogenetic relationship of *MaBAM4* and *MaBAM7* with the catalytically non-active *AtBAM9* and *AtBAM4* genes in *Arabidopsis* raises questions as to their specific functions in starch metabolism (**Figure 7**). As AtBAM4 was proposed to function as a facilitator or regulator of starch breakdown (Fulton et al., 2008), one possible hypothesis is that banana *MaBAM7* and/or *MaBAM4* have regulatory functions in the control of starch degradation in banana fruits. It remains, however, to be determined if the three genes *MaBAM6, MaBAM4* and *MaBAM7* do encode catalytically active proteins or if some of them have a different function.

Finally, all *DBE* genes were stably expressed in banana fruits which confirms absence of differential expression of the previously identified ISA3 (*MaDBE4*) banana gene and is coherent with an overall stable DBE activity in banana fruits (Bierhals et al., 2004). In addition to AMY, BAM and DBE, other enzymes such as alpha-glucan water dikinases, disproportionating enzyme 2 and starch phosphorylases are also involved in the breakdown of starch (Streb and Zeeman, 2012). Further characterization of additional starch metabolism genes has still to be performed for a more complete understanding of starch metabolism during banana ripening.

## Conclusion

We propose that within the six analyzed gene families, at least eight starch synthesis genes (*MaAGP1, MaAGP6, MaAGP7, MaSS3, MaSS8, MaSS10, MaGBSS1, MaGBSS2*), four amylases-encoding genes (*MaAMY12, MaBAM4, MaBAM6, MaBAM7*), three SBE and three DBE encoding genes, are major players involved in the starch metabolism of mature green and/or ripening banana fruits. Starch metabolism is mostly studied in *Arabidopsis* leaves, grass grains and potato tubers. Comprehensive studies including proteomics approaches in banana fruits should also improve our understanding of starch metabolism in plants and its evolution.

## Author Contributions

CJ and NY performed phylogenetic and duplication analysis; CJ, CC, and NY performed transcriptomic experiments and analysis; OG, JR, AG, DM-A-M designed, performed and analyzed fruit physiological experiments; CJ and NY designed the project and wrote the paper.

## Conflict of Interest Statement

The authors declare that the research was conducted in the absence of any commercial or financial relationships that could be construed as a potential conflict of interest.

The reviewer FG-H and handling Editor declared their shared affiliation, and the handling Editor states that the process nevertheless met the standards of a fair and objective review.

## Footnotes

1 http://banana-genome.cirad.fr/metabolic_pathway

2 http://www.greenphyl.org/cgi-bin/index.cgi

3 http://tree.bio.ed.ac.uk/software/figtree/

4 http://chibba.agtec.uga.edu/duplication/index/downloads

5 http://genomevolution.org/CoGe/SynMap.pl

6 https://genomevolution.org/CoGe/SynFind.pl

7 http://banana-genome.cirad.fr

8 http://bar.utoronto.ca/
