# Supplementary material for: Lineage-Specific Evolutionary Histories and Regulation of Major Starch Metabolism Genes during Banana Ripening
Source: Front Plant Sci. 2016 Dec 2;7:1778. doi: 10.3389/fpls.2016.01778 (PMC5133247; doi:10.3389/fpls.2016.01778)
Supplement: Supplementary file 1 [file Presentation_1.pdf]

SUPPLEMENTARY INFORMATION

Supplementary Method S1. Determination of physicochemical parameters of banana fruits. Carbon dioxide production was measured at constant room temperature of 23°C by gas chromatography after conservation of individual banana fruits in hermetic jars during two hours (Hewlett Packard 5890A, GmbH, Waldbronn, Germany). Raw data were normalised as follows:

$$A = \frac{(V \times C)}{(0.0464)} \div \frac{W}{T}$$

where A, V, C, W and T represent respectively normalised carbon dioxide production expressed in $\mu l.Kg^{-1}.h^{-1}$, the jar free volume in litres, raw data from the chromatogram expressed in µV, fruit weight in kilograms (Kg) and the period of confinement expressed in hours (h). The constant (0.0464) corresponds to the slope of the standard curve. Fruit color was estimated through the CIE L*a*b* color space system using a Minolta Chroma Meter CR-200 (Minolta, Roissy, France). L*, a* and b* values represent lightness of color from 0 (black) to 100 (diffuse white), its position between green (-300) and red (+300) and its position between blue (-300) and yellow (+300), respectively. Pulp firmness and peel hardness were measured using a TA-XT2 penetrometer as described by Chillet et al., 2008. All these parameters were determined for five biological replicates per condition.

The starch content (g/100 g of flour) was computed as follows:

$$Starch\% = \frac{(Abs \times Slope \times 0.9)}{(1000 \times DM)} \times \frac{100}{0.5} \times \frac{10}{0.05} \times 100 - FG$$

where Starch% is the percentage of starch on dry weight basis (g starch per 100g dried product); Abs is the absorbance at 510 nm against blank; Slope is the slope of the calibration curve daily obtained with a glucose solution; 0.9 is the conversion factor between starch and glucose; $1000 \times DM$ is the conversion of sample dry weight from mg to g (after correction with it residual moisture content); $\frac{100}{0.5}$ is the ratio between the total volume (100 ml) and the volume of test sample used (0.5 ml) after alpha-amylase attack; $\frac{10}{0.05}$ is the ratio between the total volume (10 ml) at amyloglucosidase action stage and the volume (0.05 ml) used for final measuring of glucose (glucose-oxidase/peroxidase/4-aminoantipirine activity); 100 is the

result given in percentage and FG is the percentage of free glucose (g free glucose per 100g dried product).

The free glucose content (g/100 g of flour) was computed as follows:

$$FG = \frac{(A \times Slope \times 1000)}{DM}$$

where *FG* is the free glucose content on dry basis, *A* is the absorbance measured for free glucose, *Slope* is the slope of the standard curve (in µg.ml^{-1}), *1000* is the conversion between mg to g and *DM* is the amount of dried sample (in mg).

Supplementary Table S1. The 12 plant predicted proteomes used in the study

Plant species	Protein number	Genome version	Link and reference
Musa acuminata	36,542	Version 1	http://banana-genome.cirad.fr (D'Hont *et al.*, 2012)
Oryza sativa	39,045	MSU version 7	http://rice.plantbiology.msu.edu (Ouyang *et al.*, 2007)
Brachypodium distachyon	26,552	Version 1.2	http://www.brachypodium.org (International Brachypodium Initiative, 2010)
Sorghum bicolor	34,009	Version 1.4	http://genome.jgi-psf.org/Sorbi1/Sorbi1.home.html (Paterson *et al.*, 2009)
Zea mays	38,915	Version 5b.60	http://www.maizesequence.org/index.html (Schnable *et al.*, 2009)
Phoenix dactylifera	41,660	Version 1	http://www.kacst.edu.sa/en/depts/jcg/home/Pages/default.aspx (Al-Mssallem *et al.*, 2013)
Arabidopsis thaliana	27,417	TAIR version 10	http://www.arabidopsis.org (Arabidopsis Genome Initiative, 2000)
Vitis vinifera	26,346	Version 1	http://www.genoscope.cns.fr/spip/Vitis-vinifera-whole-genome.html (Jaillon *et al.*, 2007)
Solanum lycopersicum	34,728	ITAG Release 2.3	http://solgenomics.net/organism/Solanum_lycopersicum/genome (The Tomato Genome Consortium, 2012)
Solanum tuberosum	39.031	Version 3.4	http://solanaceae.plantbiology.msu.edu/pgsc_download.shtml (The Potato Genome Consortium, 2011)
Prunus persica	28,689	Version 1.0	http://www.phytozome.org/peach (Verde *et al.*, 2013)
Fragaria vesca	34,894	Version 2.0	http://www.strawberrygenome.org (Shulaev *et al.*, 2011)

Supplementary Table S2. Protein reference list used for sequence clustering

Gene family	Plant species	Locus identifier	Gene name	Protein identifier	Source
AGPase	*Arabidopsis thaliana*	At1g05610	*AtAPS2*	Q7YKW3	Uniprot
AGPase	*Arabidopsis thaliana*	At1g27680	*AtAPL2*	P55230	Uniprot
AGPase	*Arabidopsis thaliana*	At2g21590	*AtAPL4*	Q9SIK1	Uniprot
AGPase	*Arabidopsis thaliana*	At4g39210	*AtAPL3*	P55231	Uniprot
AGPase	*Arabidopsis thaliana*	At5g19220	*AtADG2*	P55229	Uniprot
AGPase	*Arabidopsis thaliana*	At5g48300	*AtAPS1*	P55228	Uniprot
AGPase	*Zea mays*	GRMZM2G027955_P01	*ZmAGP1*	NP_001105717	GenBank
AGPase	*Zea mays*	GRMZM2G429899_P01	*ZmSH2*	NP_001121104	GenBank
AGPase	*Zea mays*	GRMZM2G163437_P01	*ZmL2*	AAK69628	GenBank
AGPase	*Zea mays*	GRMZM2G068506_P01	*ZmBT2*	NP_001105038	GenBank
AGPase	*Zea mays*	GRMZM2G106213_P01	*ZmAGP2*	NP_001105178	GenBank
AGPase	*Zea mays*	GRMZM2G391936_P01	*ZmAPL1*	NP_001106017	GenBank
AGPase	*Zea mays*	GRMZM2G144002_P01	*ZmAPL4*	NP_001106058	GenBank
AGPase	*Oryza sativa*	Os08g25734	*OsAPS1*	NP_001061603	GenBank
AGPase	*Oryza sativa*	Os09g12660	*OsAPS2*	NP_001062808	GenBank
AGPase	*Oryza sativa*	Os03g52460	*OsAPL1*	NP_001051184	GenBank
AGPase	*Oryza sativa*	Os01g44220	*OsAPL2*	NP_001043654	GenBank
AGPase	*Oryza sativa*	Os05g50380	*OsAPL3*	NP_001056424	GenBank
AGPase	*Oryza sativa*	Os07g13980	*OsAPL4*	NP_001059276	GenBank
AGPase	*Sorghum bicolor*	Sb03g028850	*SbSH2*	AAB94012	GenBank
AGPase	*Solanum tuberosum*	PGSC0003DMP400015875	*StGLGL3*	P55243	Uniprot
AGPase	*Solanum tuberosum*	PGSC0003DMP400027938	*StGLGL2*	P55242	Uniprot
AGPase	*Solanum tuberosum*	PGSC0003DMP400054142	*StGLGS*	P23509	Uniprot
GBSS	*Arabidopsis thaliana*	At1g32900	*AtWAXY*	Q9MAQ0	Uniprot
GBSS	*Zea mays*	GRMZM2G024993_P02	*ZmWAXY*	P04713	Uniprot
GBSS	*Oryza sativa*	Os06g04200	*OsWAXY*	Q0DEV5	Uniprot
GBSS	*Orysa sativa*	Os07g22930	*OsGBSS2*	AU030570	GenBank
GBSS	*Solanum tuberosum*	PGSC0003DMP400021398	*StWAXY*	Q00775	Uniprot
SS	*Arabidopsis thaliana*	At1g11720	*AtSS3*	F4IAG2	Uniprot
SS	*Arabidopsis thaliana*	At3g01180	*AtSS2*	Q9MAC8	Uniprot
SS	*Arabidopsis thaliana*	At4g18240	*AtSS4*	Q0WVX5	Uniprot
SS	*Arabidopsis thaliana*	At5g24300	*AtSS1*	Q9FNF2	Uniprot
SS	*Oryza sativa*	Os02g51070	*OsSSII-2*	Q6Z2T8	Uniprot
SS	*Oryza sativa*	Os06g12450	*OsSSII-3*	Q0DDE3	Uniprot
SS	*Oryza sativa*	Os10g30156.	*OsSSII-1*	Q7XE48	Uniprot
SS	*Solanum tuberosum*	PGSC0003DMP400032329	*StSS1*	P93568	Uniprot
SS	*Solanum tuberosum*	PGSC0003DMP400002384	*StSSII*	Q43847	Uniprot
SBE	*Arabidopsis thaliana*	At2g36390	*AtSBE2.1*	O23647	Uniprot
SBE	*Arabidopsis thaliana*	At5g03650	*AtSBE2.2*	Q9LZS3	Uniprot
SBE	*Arabidopsis thaliana*	At3g20440	*AtBE1*	D2WL32	Uniprot
SBE	*Zea mays*	GRMZM2G032628_P01	*ZmBEII*	Q08047	Uniprot
SBE	*Oryza sativa*	Os06g51084	*OsSBE1*	Q01401	Uniprot
DBE	*Arabidopsis thaliana*	At2g39930	*AtISA1*	O04196	Uniprot
DBE	*Arabidopsis thaliana*	At1g03310	*AtISA2*	Q8L735	Uniprot
DBE	*Arabidopsis thaliana*	At4g09020	*AtISA3*	Q9M0S5	Uniprot
AMY	*Arabidopsis thaliana*	At1g69830	*AtAMY3*	Q94A41	Uniprot

AMY	*Arabidopsis thaliana*	At1g76130	*AtAMY2*	Q8LFG1	Uniprot
AMY	*Arabidopsis thaliana*	At4g25000	*AtAMY1*	Q8VZ56	Uniprot
AMY	*Oryza sativa*	Os01g25510	*OsAMY1B*	Q0JMV4	Uniprot
AMY	*Oryza sativa*	Os02g52710	*OsAMY1.1*	P17654	Uniprot
AMY	*Oryza sativa*	Os06g49970	*OsAMY2A*	Q0D9J1	Uniprot
AMY	*Oryza sativa*	Os08g36900	*OsAMY1.4*	P27934	Uniprot
AMY	*Oryza sativa*	Os08g36910	*OsAMY1.3*	P27933	Uniprot
AMY	*Oryza sativa*	Os09g28400	*OsAMY1.2*	P27932	Uniprot
AMY	*Oryza sativa*	Os09g28420	*OsAMY1.6*	P27937	Uniprot
BAM	*Arabidopsis thaliana*	At2g32290	*AtBAM6*	Q8L762	Uniprot
BAM	*Arabidopsis thaliana*	At2g45880	*AtBAM7*	O80831	Uniprot
BAM	*Arabidopsis thaliana*	At3g23920	*AtBAM1*	Q9LIR6	Uniprot
BAM	*Arabidopsis thaliana*	At4g00490	*AtBAM2*	O65258	Uniprot
BAM	*Arabidopsis thaliana*	At4g15210	*AtBAM5*	P25853	Uniprot
BAM	*Arabidopsis thaliana*	At4g17090	*AtBAM3*	O23553	Uniprot
BAM	*Arabidopsis thaliana*	At5g18670	*AtBAM9*	Q8VYW2	Uniprot
BAM	*Arabidopsis thaliana*	At5g45300	*AtBAM8*	Q9FH80	Uniprot
BAM	*Arabidopsis thaliana*	At5g55700	*AtBAM4*	Q9FM68	Uniprot

Supplementary Table S3. List of primer sequences used for qRT-PCR experiments and characteristics of corresponding amplicons

Gene name	Primer ID	Primer sequence (5'-3')	Amplicon size (bp)	E (±SE)*
MaACT2	Ma1067F	GGAGTCTGGTCCTGCTATCG	147	1.981 (±0.00494)
	Ma1067R	AAAGTCCGATGCAACAAAGC		
MaAGP1	Ma1149F	CTCGGGCTTTGTTCATGTTC	125	2.01 (±0.00992)
	Ma1149R	TGTGCTCCCACAAACATCAT		
MaAGP4	Ma1027F	AATGCCAGTTCTCGACGTTT	132	1.946 (±0.00523)
	Ma1027R	CTGCATTAGGGCAGCTGATT		
MaAGP5	Ma1202F	ATGATGGGTGCTGACGACTA	90	1.856 (±0.0229)
	Ma1202R	TGTGTTCTCTCCCACTCCAA		
MaAGP6	Ma1190F	GCGAGGGCTTCTACATACGA	132	2.001 (±0.0106)
	Ma1190R	GCCGTAGCATCCTCTCCTC		
MaAGP7	Ma1188F	CGAAGGATTCAGCAAGGAAG	129	1.896 (±0.0265)
	Ma1188R	TGGGCACATACAAAGCTATCA		
MaSS3	Ma1018F	CACTTCATCCACACCTTCCA	88	1.922 (±0.00442)
	Ma1018R	CGACAGATTCATTTCGTTCG		
MaSS8	Ma1205F	CTTGGCTGAGACGAGGACTT	123	1.918 (±0.0209)
	Ma1205R	TGCCTCAACACCTACAGGAAC		
MaSS10	Ma1208F	CGTGCCGAATGGTATAGCTC	99	1.962 (±0.00546)
	Ma1208R	ACGGGAATGGTTCAACTCAC		
MaGBSS1	Ma1007F	AAGATCGGCTTCAGACCAGA	82	1.969 (±0.00768)
	Ma1007R	GGGACTCTGAGGATTGTCCA		
MaGBSS2	Ma1073F	GGAGCTGCTGGTAGTGAAGC	87	2.08 (±0.0459)
	Ma1073R	TAGTCGCTTTCAGGGAGTGG		
MaGBSS3	Ma1110F	ATTGGAGCAACAGCTTGCAT	86	2.07 (±0.00164)
	Ma1110R	CATGAGCCAGAGGAACATTG		
MaGBSS4	Ma1109F	GTGAAGCAATGCACCACATT	85	NA
	Ma1109R	TGTTGTGAATGGCGTAGGTG		
MaSBE1	Ma1227F	CATCGGAAGCTAGGTCAGGA	114	1.889 (±0.0333)
	Ma1227R	TCCAAGATTCCCAGGTTGAC		
MaSBE2	Ma1233F	TCCTGGAACGTTGGTTTCTC	89	2.008 (±0.0055)
	Ma1233R	TGGTGATGTGTTTGGTCGTT		
MaSBE3	Ma1235F	AGGCCTCGCTCTTTCCTAGT	92	1.957 (±0.00527)
	Ma1235R	ATGCACAACCCAGCAATCTT		
MaSBE4	Ma1238F	GACTTGCATAGTGGCATTCG	100	1.939 (±0.0137)
	Ma1238R	CTGAGGCGACTACGGGACTA		
MaSBE5	Ma1243F	ACCAACCAGCCTCTCATTTG	93	1.952 (±0.0109)
	Ma1243R	GAACCGCAGTTGTTTATGCTC		
MaDBE1	Ma1211F	TGTGATCCAAATGTTCAGAAGG	101	1.985 (±0.054)
	Ma1211R	AGGCACAAAGTTCAATCCACA		
MaDBE3	Ma1218F	CCTTCAACACAAGCCATCTG	99	1.902 (±0.0251)
	Ma1218R	CGTAGGGCACAGGCTTACTC		
MaDBE4	Ma1224F	CGAAGGTGTACCATTCAGCA	83	1.899 (±0.00424)
	Ma1224R	CACAGGATCATGGCTTTGC		
MaBAM4	Ma1020F	CATGAACCTTCAGACATTCAGG	145	1.961 (±0.00501)
	Ma1020R	CAGTGTAGCGATTCTACTGCATC		
MaBAM6	Ma1030F	ATCCGCTGCCTGATGACTAC	114	2.083 (±0.00823)
	Ma1030R	ATTGTTTGCTATGCGTGCAG		
MaBAM7	Ma1034F	TGGCAGCAGAAGAGATATCCA	100	1.921 (±0.00568)
	Ma1034R	CATACGTTCGGCAAATCCTT		
MaAMY1	Ma1155F	CTTGTGCCAGACTGCGTATC	116	1.981 (±0.0356)
	Ma1155R	ATGCTGCAAGAATCGACAAC		
MaAMY2	Ma1157F	CATGACCCATTGCCAATCTT	99	NA
	Ma1157R	CCATAGTCGCTCCAAGATCA		
MaAMY3	Ma1159F	CTTGTGCCAAACTGCGTATC	131	1.998 (±0.0163)
	Ma1159R	CTCGCTCCGATCTCTATGCT		
MaAMY4	Ma1161F	ACCAAAGTCGACTCGGTCA	129	2.097 (±0.064)
	Ma1161R	TATAGCCAACGCTGGAGTCA		
MaAMY5	Ma1194F	TTTCCCTTGTTGGCCGTGAG	134	2.03 (±0.0333)

	Ma1194R	AGCACCATGGAACTCGATTATTTC		
MaAMY8	Ma1198F	AACTAGCCCTTGGCGGTTAC	115	1.957 (±0.0589)
	Ma1198R	CTACGTCGAACAAACGCAGC		
MaAMY12	Ma1153F	ATAGCTGAAACCTCGGAGCA	129	2.014 (±0.0373)
	Ma1153R	TGGGTTGTAGCAGCTGAAGG		

NA, not available value *i.e.* gene not or very weakly expressed.

*Efficiency (E) and mean squared error (±SE) for primer pair in pulp tissue.

Supplementary Table S4. Isoform numbers of starch metabolism-related gene families in 12 plant genomes

	Plant species											
	Monocots						Dicots					
Gene family	**Ma**	Os	Bd	Sb	Zm	Pd	At	Vv	Sl	St	Pp	Fv
AGPase	**7**	6	5	7	7	7	6	6	6	5	6	5
SS	**10**	9	7	8	7	8	5	6	6	6	6	6
GBSS	**4**	2	2	2	2	1	1	2	1	1	2	2
SBE	**4**	3	3	3	4	3	2	2	2	2	2	3
DBE	**4**	3	3	3	3	3	3	3	3	3	3	3
BAM	**13**	10	11	10	13	10	9	9	8	8	10	9
AMY	**13**	10	5	11	7	4	3	8	5	3	5	4
Total	**46**	37	30	38	36	30	24	31	26	23	29	26

Ma, *Musa acuminata*; Os, *Oryza sativa*; Bd, *Brachypodium distachyon*; Sb, *Sorghum bicolor*; Zm, *Zea mays* ; Pd, *Phoenix dactylifera*; At, *Arabidopsis thaliana*, Vv, *Vitis vinifera*; Sl, *Solanum lycopersicum*; St, *Solanum tuberosum*; Pp, *Prunus persica*; Fv, *Fragaria vesca* ; AGPase, ADP-glucose pyrophosphorylase ; SS, soluble starch synthase ; GBSS, granule-bound starch synthase ; SBE, starch branching enzyme ; DBE, debranching enzyme ; AMY, α-amylase and BAM, β-amylase.

Supplementary Table S5. Table of starch metabolism-related genes identified in banana

**Gene family**	**Gene order**[*]	**Gene name**	**Locus identifier Genome version 1**[$]
AGPase	1	*MaAGP1*	GSMUA Achr1G09400 001
AGPase	2	*MaAGP2*	GSMUA Achr1G11820 001
AGPase	3	*MaAGP3*	GSMUA Achr3G21710 001
AGPase	4	*MaAGP4*	GSMUA Achr4G02930 001
AGPase	5	*MaAGP5*	GSMUA Achr4G10620 001
AGPase	6	*MaAGP6*	GSMUA Achr6G27190 001
AGPase	7	*MaAGP7*	GSMUA Achr9G06920 001
SS	1	*MaSS1*	GSMUA Achr1G24640 001
SS	2	*MaSS2*	GSMUA Achr1G24650 001
SS	3	*MaSS3*	GSMUA Achr3G03290 001
SS	4	*MaSS4*	GSMUA Achr3G16340 001
SS	5	*MaSS5*	GSMUA Achr4G10220 001
SS	6	*MaSS6*	GSMUA Achr5G00700 001
SS	7	*MaSS7*	GSMUA Achr6G23190 001
SS	8	*MaSS8*	GSMUA Achr8G24090 001
SS	9	*MaSS9*	GSMUA Achr8G27560 001
SS	10	*MaSS10*	GSMUA Achr11G18560 001
GBSS	1	*MaGBSS1*	GSMUA Achr1G06140 001
GBSS	2	*MaGBSS2*	GSMUA Achr1G08190 001
GBSS	3	*MaGBSS3*	GSMUA Achr1G16610 001
GBSS	4	*MaGBSS4*	GSMUA Achr9G20780 001
SBE	1	*MaSBE1*	GSMUA Achr4G04690 001
SBE	2	*MaSBE2*	GSMUA Achr5G16760 001
SBE	3	*MaSBE3*	GSMUA Achr6G07530 001
SBE	4	*MaSBE4*	GSMUA Achr6G13530 001
SBE	5	*MaSBE5*	GSMUA Achr6G24120 001
DBE	1	*MaDBE1*	GSMUA Achr6G16190 001
DBE	2	*MaDBE2*	GSMUA Achr6G16200 001
DBE	3	*MaDBE3*	GSMUA Achr9G14020 001
DBE	4	*MaDBE4*	GSMUA Achr9G14980 001
AMY	1	*MaAMY1*	GSMUA Achr1G02190 001
AMY	2	*MaAMY2*	GSMUA Achr1G02210 001
AMY	3	*MaAMY3*	GSMUA Achr1G02230 001
AMY	4	*MaAMY4*	GSMUA Achr3G07130 001
AMY	5	*MaAMY5*	GSMUA Achr3G07190 001
AMY	6	*MaAMY6*	GSMUA Achr5G08130 001
AMY	7	*MaAMY7*	GSMUA Achr5G08290 001
AMY	8	*MaAMY8*	GSMUA Achr5G08340 001
AMY	9	*MaAMY9*	GSMUA Achr5G10560 001
AMY	10	*MaAMY10*	GSMUA Achr7G18920 001
AMY	11	*MaAMY11*	GSMUA Achr7G26940 001
AMY	12	*MaAMY12*	GSMUA Achr8G04140 001
AMY	13	*MaAMY13*	GSMUA Achr10G30130 001
BAM	1	*MaBAM1*	GSMUA Achr1G17440 001
BAM	2	*MaBAM2*	GSMUA Achr2G04590 001
BAM	3	*MaBAM3*	GSMUA Achr2G14060 001
BAM	4	*MaBAM4*	GSMUA Achr3G08860 001
BAM	5	*MaBAM5*	GSMUA Achr3G27320 001
BAM	6	*MaBAM6*	GSMUA Achr4G17840 001

BAM	7	*MaBAM7*	GSMUA Achr5G08040 001	
BAM	8	*MaBAM8*	GSMUA Achr5G10120 001	
BAM	9	*MaBAM9*	GSMUA Achr6G05840 001	
BAM	10	*MaBAM10*	GSMUA Achr7G11100 001	
BAM	11	*MaBAM11*	GSMUA Achr8G14930 001	
BAM	12	*MaBAM12*	GSMUA Achr10G09320 001	
BAM	13	*MaBAM13*	GSMUA AchrUn randomG28890 001	

[*]Gene order in Fig. 4A; [$]D'Hont et al., 2012

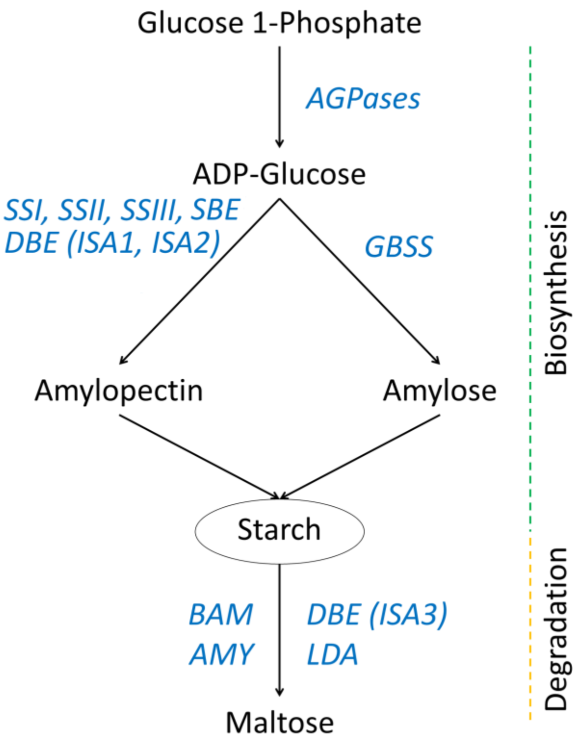

Supplementary Figure S1. Schematic and simplified view of starch biosynthesis and degradation

Major gene families involved are indicated in blue. Adenosine diphosphoglucose (ADP-glucose), the glucose donor for starch synthesis is synthesized by ADP-glucose pyrophosphorylases (AGPases) that use adenosine triphosphate (ATP) and glucose-1-phosphate as substrates. Starch synthases (SS) catalyze the transfer of glucose from ADP-glucose to non-reducing ends of glucose chains via α-1,4-bonds. Synthesis of amylopectin requires the elongation of glucan chains by SSI-IV soluble starch synthases and the introduction of branch points by SBE starch branching enzymes. ISA1 and ISA2 debranching enzymes (DBE) are proposed to be involved in the removal of misplaced amylopectin branches. Granule-bound starch synthases (GBSS) synthesizes very long glucan chains that are found mainly in amylose. Starch breakdown is dependent on the phosphorylation status of glucans at the surface of starch granules. The exo-acting β-amylases (BAM) release β-maltose from exposed non-reducing ends of chains. Endo-acting α-amylases (AMY) hydrolyze α-1,4-linked glucose chains and release a mixture of soluble linear and branched oligosaccharides. Debranching enzymes ISA3 and pullulanases/limit dextrinases (LDA) are involved in breakdown of branched amylopectin chains. Based on reviews by (Zeeman et al., 2010; Streb and Zeeman, 2012) .

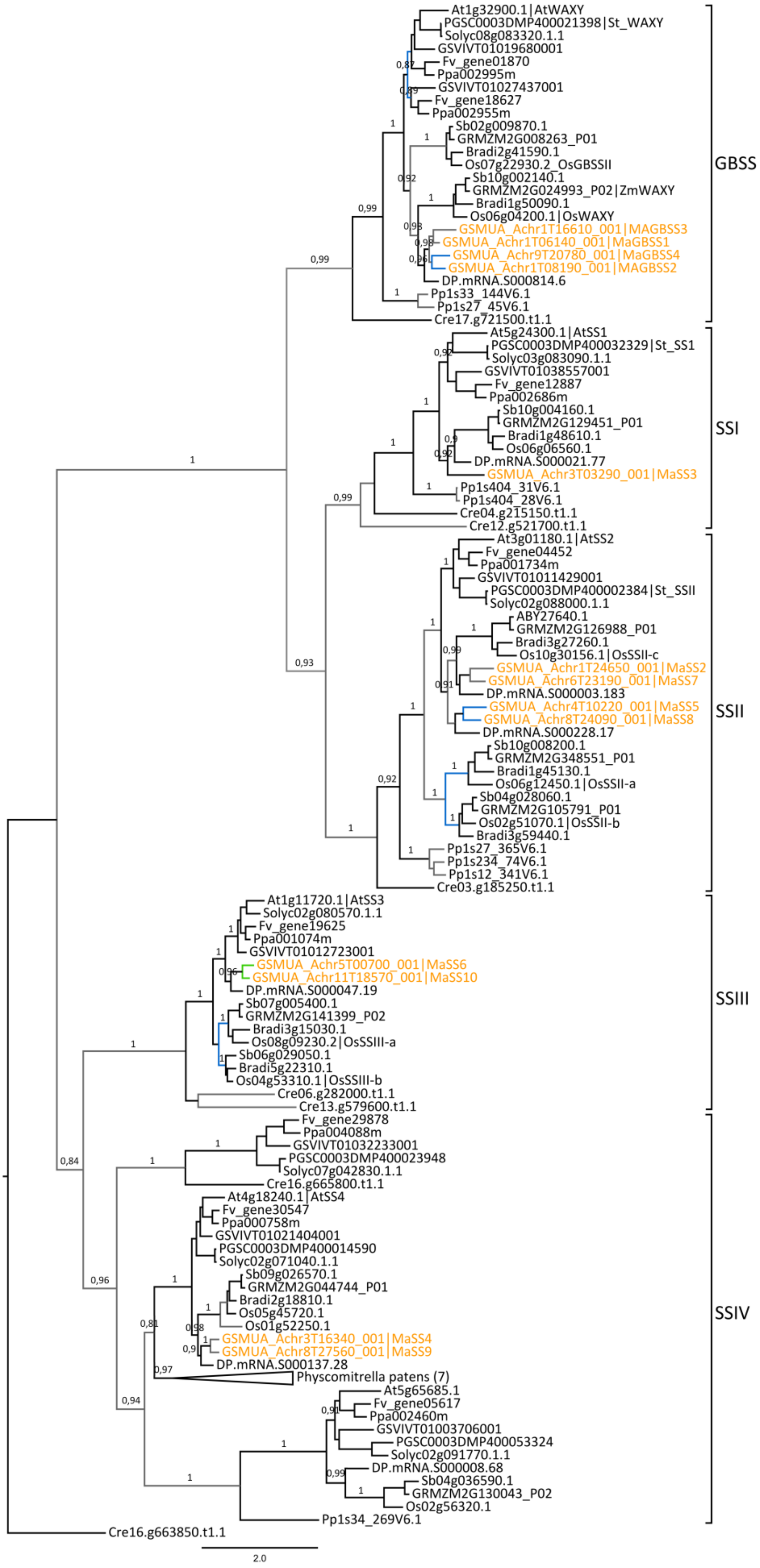

At1g32900.1|AtWAXY
PGSC0003DMP400021398|St_WAXY
Solyc08g083320.1.1
GSVIVT01019680001
Fv_gene01870
Ppa002995m
GSVIVT01027437001
Fv_gene18627
Ppa002955m
Sb02g009870.1
GRMZM2G008263_P01
Bradi2g41590.1
Os07g22930.2_OsGBSSII
Sb10g002140.1
GRMZM2G024993_P02|ZmWAXY
Bradi1g50090.1
Os06g04200.1|OsWAXY
GSMUA_Achr1T16610_001|MAGBSS3
GSMUA_Achr1T06140_001|MaGBSS1
GSMUA_Achr9T20780_001|MaGBSS4
GSMUA_Achr1T08190_001|MAGBSS2
DP.mRNA.S000814.6
Pp1s33_144V6.1
Pp1s27_45V6.1
Cre17.g721500.t1.1
GBSS
At5g24300.1|AtSS1
PGSC0003DMP400032329|St_SS1
Solyc03g083090.1.1
GSVIVT01038557001
Fv_gene12887
Ppa002686m
Sb10g004160.1
GRMZM2G129451_P01
Bradi1g48610.1
Os06g06560.1
DP.mRNA.S000021.77
GSMUA_Achr3T03290_001|MaSS3
Pp1s404_31V6.1
Pp1s404_28V6.1
Cre04.g215150.t1.1
Cre12.g521700.t1.1
SSI
At3g01180.1|AtSS2
Fv_gene04452
Ppa001734m
GSVIVT01011429001
PGSC0003DMP400002384|St_SSII
Solyc02g088000.1.1
ABY27640.1
GRMZM2G126988_P01
Bradi3g27260.1
Os10g30156.1|OsSSII-c
GSMUA_Achr1T24650_001|MaSS2
GSMUA_Achr6T23190_001|MaSS7
DP.mRNA.S000003.183
GSMUA_Achr4T10220_001|MaSS5
GSMUA_Achr8T24090_001|MaSS8
DP.mRNA.S000228.17
Sb10g008200.1
GRMZM2G348551_P01
Bradi1g45130.1
Os06g12450.1|OsSSII-a
Sb04g028060.1
GRMZM2G105791_P01
Os02g51070.1|OsSSII-b
Bradi3g59440.1
Pp1s27_365V6.1
Pp1s234_74V6.1
Pp1s12_341V6.1
Cre03.g185250.t1.1
SSII
At1g11720.1|AtSS3
Solyc02g080570.1.1
Fv_gene19625
Ppa001074m
GSVIVT01012723001
GSMUA_Achr5T00700_001|MaSS6
GSMUA_Achr11T18570_001|MaSS10
DP.mRNA.S000047.19
Sb07g005400.1
GRMZM2G141399_P02
Bradi3g15030.1
Os08g09230.2|OsSSIII-a
Sb06g029050.1
Bradi5g22310.1
Os04g53310.1|OsSSIII-b
Cre06.g282000.t1.1
Cre13.g579600.t1.1
SSIII
Fv_gene29878
Ppa004088m
GSVIVT01032233001
PGSC0003DMP400023948
Solyc07g042830.1.1
Cre16.g665800.t1.1
At4g18240.1|AtSS4
Fv_gene30547
Ppa000758m
GSVIVT01021404001
PGSC0003DMP400014590
Solyc02g071040.1.1
Sb09g026570.1
GRMZM2G044744_P01
Bradi2g18810.1
Os05g45720.1
Os01g52250.1
GSMUA_Achr3T16340_001|MaSS4
GSMUA_Achr8T27560_001|MaSS9
DP.mRNA.S000137.28
Physcomitrella patens (7)
At5g65685.1
Fv_gene05617
Ppa002460m
GSVIVT01003706001
PGSC0003DMP400053324
Solyc02g091770.1.1
DP.mRNA.S000008.68
Sb04g036590.1
GRMZM2G130043_P02
Os02g56320.1
Pp1s34_269V6.1
SSIV
Cre16.g663850.t1.1
2.0

Supplementary Figure S2. Maximum likelihood phylogenetic tree of starch synthase (SS and GBSS) predicted proteins.

Only supports (aLRT statistics with SH–like procedure) of main branches are indicated. *Chlamydomonas reinhardtii* (Cre), *Physcomitrella patens* (Pp), Grapevine (GSVIV), tomato (Solyc), potato (PGSC), woodland strawberry (Fv), *Arabidopsis* (At), peach (Ppa), *Brachypodium* (Bradi), rice (Os), maize (GRMZM), sorghum (Sb), date palm (DP) and banana (Ma) identifiers are indicated (in brown for banana). Branches are colored according to gene duplication modes: WGD (blue), segmental (green), tandem/proximal (red) and unknown (grey). Black branches correspond to speciation events. The scale bar corresponds to estimated amino acid substitutions per site. Sequences were grouped using the tree topology and previously published classifications (Patron and Keeling, 2005; Leterrier et al., 2008).

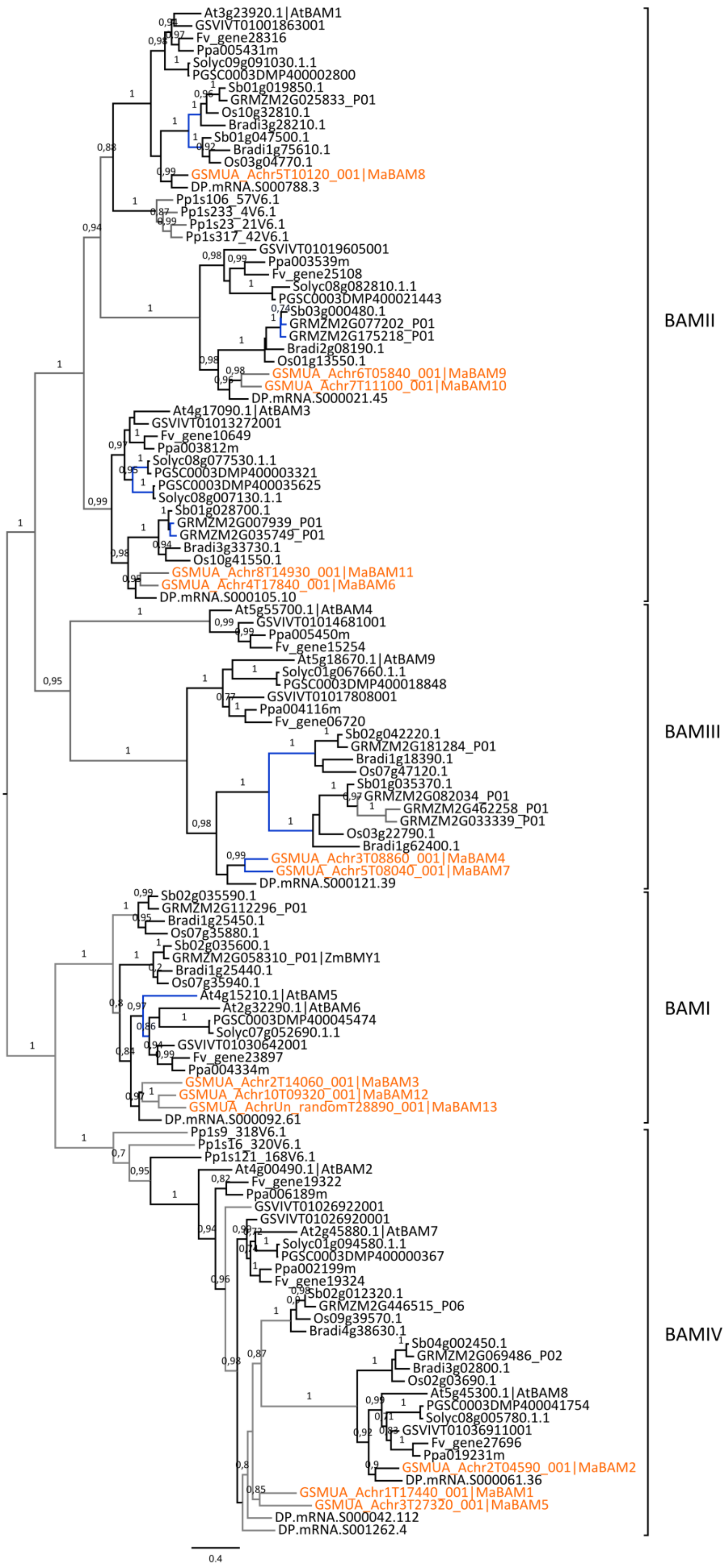

At3g23920.1|AtBAM1
GSVIVT01001863001
Fv_gene28316
Ppa005431m
Solyc09g091030.1.1
PGSC0003DMP400002800
Sb01g019850.1
GRMZM2G025833_P01
Os10g32810.1
Bradi3g28210.1
Sb01g047500.1
Bradi1g75610.1
Os03g04770.1
GSMUA_Achr5T10120_001|MaBAM8
DP.mRNA.S000788.3
Pp1s106_57V6.1
Pp1s233_4V6.1
Pp1s23_21V6.1
Pp1s317_42V6.1
GSVIVT01019605001
Ppa003539m
Fv_gene25108
Solyc08g082810.1.1
PGSC0003DMP400021443
Sb03g000480.1
GRMZM2G077202_P01
GRMZM2G175218_P01
Bradi2g08190.1
Os01g13550.1
GSMUA_Achr6T05840_001|MaBAM9
GSMUA_Achr7T11100_001|MaBAM10
DP.mRNA.S000021.45
At4g17090.1|AtBAM3
GSVIVT01013272001
Fv_gene10649
Ppa003812m
Solyc08g077530.1.1
PGSC0003DMP400003321
PGSC0003DMP400035625
Solyc08g007130.1.1
Sb01g028700.1
GRMZM2G007939_P01
GRMZM2G035749_P01
Bradi3g33730.1
Os10g41550.1
GSMUA_Achr8T14930_001|MaBAM11
GSMUA_Achr4T17840_001|MaBAM6
DP.mRNA.S000105.10
At5g55700.1|AtBAM4
GSVIVT01014681001
Ppa005450m
Fv_gene15254
At5g18670.1|AtBAM9
Solyc01g067660.1.1
PGSC0003DMP400018848
GSVIVT01017808001
Ppa004116m
Fv_gene06720
Sb02g042220.1
GRMZM2G181284_P01
Bradi1g18390.1
Os07g47120.1
Sb01g035370.1
GRMZM2G082034_P01
GRMZM2G462258_P01
GRMZM2G033339_P01
Os03g22790.1
Bradi1g62400.1
GSMUA_Achr3T08860_001|MaBAM4
GSMUA_Achr5T08040_001|MaBAM7
DP.mRNA.S000121.39
Sb02g035590.1
GRMZM2G112296_P01
Bradi1g25450.1
Os07g35880.1
Sb02g035600.1
GRMZM2G058310_P01|ZmBMY1
Bradi1g25440.1
Os07g35940.1
At4g15210.1|AtBAM5
At2g32290.1|AtBAM6
PGSC0003DMP400045474
Solyc07g052690.1.1
GSVIVT01030642001
Fv_gene23897
Ppa004334m
GSMUA_Achr2T14060_001|MaBAM3
GSMUA_Achr10T09320_001|MaBAM12
GSMUA_AchrUn_randomT28890_001|MaBAM13
DP.mRNA.S000092.61
Pp1s9_318V6.1
Pp1s16_320V6.1
Pp1s121_168V6.1
At4g00490.1|AtBAM2
Fv_gene19322
Ppa006189m
GSVIVT01026922001
GSVIVT01026920001
At2g45880.1|AtBAM7
Solyc01g094580.1.1
PGSC0003DMP400000367
Ppa002199m
Fv_gene19324
Sb02g012320.1
GRMZM2G446515_P06
Os09g39570.1
Bradi4g38630.1
Sb04g002450.1
GRMZM2G069486_P02
Bradi3g02800.1
Os02g03690.1
At5g45300.1|AtBAM8
PGSC0003DMP400041754
Solyc08g005780.1.1
GSVIVT01036911001
Fv_gene27696
Ppa019231m
GSMUA_Achr2T04590_001|MaBAM2
DP.mRNA.S000061.36
GSMUA_Achr1T17440_001|MaBAM1
GSMUA_Achr3T27320_001|MaBAM5
DP.mRNA.S000042.112
DP.mRNA.S001262.4
BAMII
BAMIII
BAMI
BAMIV
0.4

Supplementary Figure S3. Phylogenetic analysis of the BAM family.

The maximum likelihood tree of BAM predicted proteins was rooted using midpoint. Supports >0.70 (aLRT statistics with SH–like procedure) are indicated. *Physcomitrella patens* (Pp), Grapevine (GSVIV), tomato (Solyc), potato (PGSC), woodland strawberry (Fv), *Arabidopsis* (At), peach (Ppa), *Brachypodium* (Bradi), rice (Os), maize (GRMZM), Sorghum (Sb), date palm (DP) and banana (GSMUA) identifiers are indicated (in brown for banana). Branches are colored according to gene duplication modes: WGD (blue), segmental (green), tandem/proximal (red) and unknown (grey). Black branches correspond to speciation events. The scale bar corresponds to estimated amino acid substitutions per site. Sequences were grouped using the tree topology and a previous classification (Fulton et al., 2008).

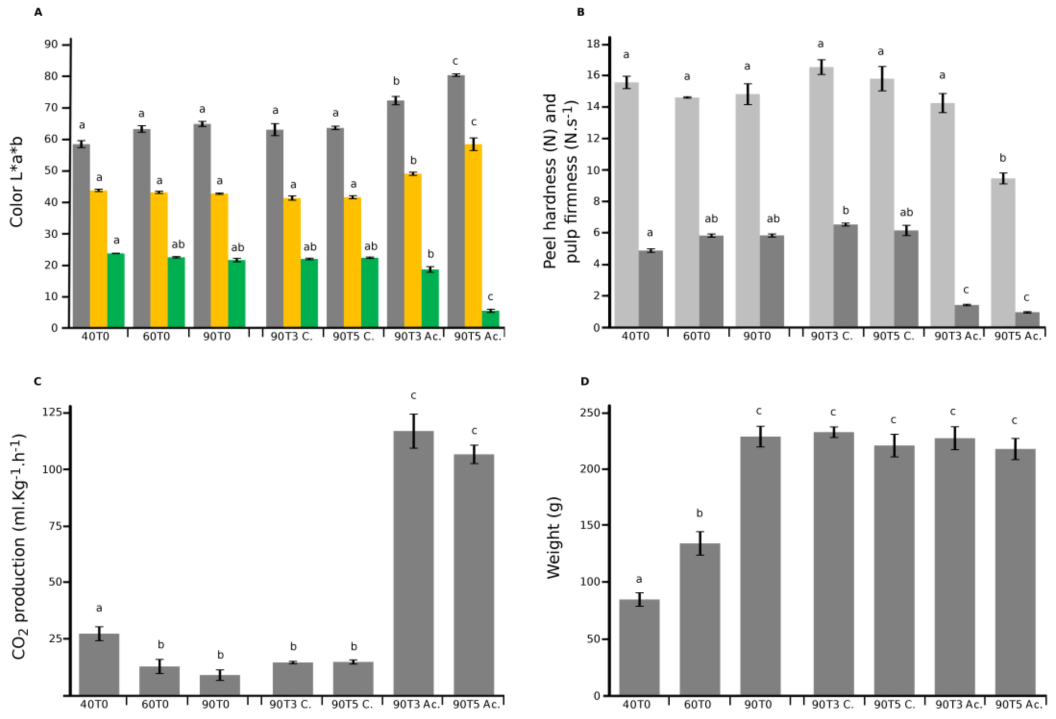

Supplementary Figure S4. Changes of physicochemical parameters of banana fruits during green fruit development and ripening.

(A) Switch from green banana fruits to yellow banana fruits. Banana fruit colors were estimated through the color space CIE L*a*b*. Green, yellow and grey histograms represent - a, b and L values without unit, respectively. **(B)** Rheological properties of banana fruits before and after acetylene treatment. Light grey and dark grey histograms represent peel hardness (expressed in Newton (N)) and pulp firmness (expressed in $N.s^{-1}$), respectively. **(C)** Respiration of banana fruits before and after acetylene treatment. Carbon dioxide productions of banana fruits are expressed in $ml.Kg^{-1}.h^{-1}$ **(D)** Filling of banana fruits during green banana fruit development. Fruit weight is expressed in grams (g). All parameters were measured individually on all fruits harvested at 40, 60 and 90 DAF immediately after harvest (40T0, 60T0 and 90T0, respectively), on fruits harvested at 90 DAF then treated 24h with acetylene and stored two or four additional days (90T3 Ac. and 90T5 Ac., respectively) and on control fruits not treated and stored three (90T3C.) and five days (90T5C.). Means, standard errors and different groups from multiple comparisons with confidence interval at 95% (ANOVA followed by Tukey's test) are indicated.

Supplementary References

Al-Mssallem I.S., Hu S., Zhang X., Lin Q., Liu W., Tan J., Yu X., Liu J., Pan L., Zhang T., Yin Y., Xin C., Wu H., Zhang G., Ba Abdullah M.M., Huang D., Fang Y., Alnakhli Y.O., Jia S., Yin A., Alhuzimi E.M., Alsaihati B.A., Al-Owayyed S.A., Zhao D., Zhang S., Al-Otaibi N.A., Sun G., Majrashi M.A., Li F., Tala, Wang J., Yun Q., Alnassar N.A., Wang L., Yang M., Al-Jelaify R.F., Liu K., Gao S., Chen K., Alkhaldi S.R., Liu G., Zhang M., Guo H. and Yu J. 2013. Genome sequence of the date palm *Phoenix dactylifera* L. *Nature Communications*, 4. doi: 10.1038/ncomms3274.

Arabidopsis Genome Initiative. (2000). Analysis of the genome sequence of the flowering plant *Arabidopsis thaliana*. *Nature* 408, 796 – 815. doi: 10.1038/35048692

D'Hont, A., Denoeud, F., Aury, J.M., Baurens, F.C., Carreel, F., Garsmeur, O., Noel, B., Bocs, S., Droc, G., Rouard, M., Da Silva, C., Jabbari, K., Cardi, C., Poulain, J., Souquet, M., Labadie, K., Jourda, C., Lengelle, J., Rodier-Goud, M., Alberti, A., Bernard, M., Correa, M., Ayyampalayam, S., Mckain, M.R., Leebens-Mack, J., Burgess, D., Freeling, M., Mbeguie, A.M.D., Chabannes, M., Wicker, T., Panaud, O., Barbosa, J., Hribova, E., Heslop-Harrison, P., Habas, R., Rivallan, R., Francois, P., Poiron, C., Kilian, A., Burthia, D., Jenny, C., Bakry, F., Brown, S., Guignon, V., Kema, G., Dita, M., Waalwijk, C., Joseph, S., Dievart, A., Jaillon, O., Leclercq, J., Argout, X., Lyons, E., Almeida, A., Jeridi, M., Dolezel, J., Roux, N., Risterucci, A.M., Weissenbach, J., Ruiz, M., Glaszmann, J.C., Quetier, F., Yahiaoui, N., and Wincker, P. (2012). The banana (*Musa acuminata*) genome and the evolution of monocotyledonous plants. Nature 488, 213-217. doi: 10.1038/nature11241.

Chillet, M., De Bellaire, L.D., Hubert, O., and Mbeguie-A-Mbeguie, D. (2008). Mechanical characterisation of banana fruits. *Fruits* 63, 51-52. doi:10.1051/fruits:2007045

Fulton, D.C., Stettler M., Mettler T., Vaughan C.K., Li J., Francisco P., Gil M., Reinhold H., Eicke S., Messerli G., *et al.* (2008). β-AMYLASE4, a noncatalytic protein required for starch breakdown, acts upstream of three active β-amylases in *Arabidopsis* chloroplasts. *The Plant Cell* 20, 1040 –1058. doi: 10.1105/tpc.107.056507.

International Brachypodium Initiative (2010). Genome sequencing and analysis of the model grass *Brachypodium distachyon*. *Nature* 463, 763–768. doi: 10.1038/nature08747

Jaillon O., Aury J.-M., Noel B., Policriti A., Clepet C., Casagrande A., Choisne N., Aubourg S., Vitulo N., Jubin C., *et al.* (2007). The grapevine genome sequence suggests ancestral hexaploidization in major angiosperm phyla. *Nature* 449, 463–467. doi: 10.1038/nature06148

Leterrier M., Holappa L., Broglie K., Beckles D. (2008). Cloning, characterisation and comparative analysis of a starch synthase IV gene in wheat, functional and evolutionary implications. *BMC Plant Biology* 8, 98. doi: 10.1186/1471-2229-8-98

Ouyang S., Zhu W., Hamilton J., Lin H., Campbell M., Childs K., Thibaud-Nissen F., Malek R.L., Lee Y., Zheng L., *et al.* (2007). The TIGR rice genome annotation resource, improvements and new features. *Nucleic Acids Research* 35, D883–D887. doi: 10.1093/nar/gkl976

Paterson A.H., Bowers J.E., Bruggmann R., Dubchak I., Grimwood J., Gundlach H., Haberer G., Hellsten U., Mitros T., Poliakov A., *et al.* (2009). The *Sorghum bicolor* genome and the diversification of grasses. *Nature* 457, 551–556. doi: 10.1038/nature07723

Patron N.J., Keeling P.J. (2005). Common evolutionary origin of starch biosynthetic enzymes in green and red algae. *Journal of Phycology* 41, 1131–1141. doi: 10.1111/j.1529-8817.2005.00135.x.

Schnable P.S., Ware D., Fulton R.S., Stein J.C., Wei F., Pasternak S., Liang C., Zhang J., Fulton L., Graves T.A., *et al.* (2009). The B73 maize genome, complexity, diversity, and dynamics. *Science* 326, 1112 –1115. doi: 10.1126/science.1178534

Shulaev V., Sargent D.J., Crowhurst R.N., Mockler T.C., Folkerts O., Delcher A.L., Jaiswal P., Mockaitis K., Liston A., Mane S.P., *et al.* (2011). The genome of woodland strawberry (*Fragaria vesca*). *Nature Genetics* 43, 109–116. doi: 10.1038/ng.740

Streb, S., and Zeeman, S.C. (2012). Starch metabolism in Arabidopsis. *The Arabidopsis book / American Society of Plant Biologists* 10**,** e0160. doi: 10.1199/tab.0160.

The Potato Genome Consortium (2011). Genome sequence and analysis of the tuber crop potato. *Nature* 475, 189–195. doi: 10.1038/nature10158

The Tomato Genome Consortium (2012). The tomato genome sequence provides insights into fleshy fruit evolution. *Nature* 485, 635–641.doi: 10.1038/nature11119

Verde I., Abbott A.G., Scalabrin S., Jung S., Shu S., Marroni F., Zhebentyayeva T., Dettori M.T., Grimwood J., Cattonaro F., *et al.* (2013). The high-quality draft genome of peach (*Prunus persica*) identifies unique patterns of genetic diversity, domestication and genome evolution. *Nature Genetics* 45, 487–494. doi : 10.1038/ng.2586

Zeeman, S.C., Kossmann, J., and Smith, A.M. (2010). Starch: its metabolism, evolution, and biotechnological modification in plants. *Annu. Rev. Plant Biol.* 61**,** 209-234. doi: 10.1146/annurev-arplant-042809-112301.
